# High density SNP and SSR-based genetic maps of two independent oil palm hybrids

**DOI:** 10.1186/1471-2164-15-309

**Published:** 2014-04-27

**Authors:** Ngoot-Chin Ting, Johannes Jansen, Sean Mayes, Festo Massawe, Ravigadevi Sambanthamurthi, Leslie Cheng-Li Ooi, Cheuk Weng Chin, Xaviar Arulandoo, Tzer-Ying Seng, Sharifah Shahrul Rabiah Syed Alwee, Maizura Ithnin, Rajinder Singh

**Affiliations:** Advanced Biotechnology and Breeding Centre, Malaysian Palm Oil Board (MPOB), No. 6, Persiaran Institusi, Bandar Baru Bangi, 43000 Kajang, Selangor Malaysia; Biometris, Wageningen University and Research Centre, P.O. Box 100, 6700 AC Wageningen, the Netherlands; Plant and Crop Sciences, Sutton Bonington Campus, University of Nottingham, Sutton Bonington, LE12 5RD Loughborough, Nottingham, UK; School of Biosciences, University of Nottingham Malaysia Campus, Jalan Broga, 43500 Selangor Semenyih, Nottingham, Malaysia; FELDA Agricultural Services Sdn. Bhd., 7th Floor, Balai Felda, Jalan Gurney 1, 54000 Kuala Lumpur, Malaysia; United Plantations Bhd., Jendarata Estate, 36009 Teluk Intan, Perak Malaysia

**Keywords:** *Elaeis guineensis*, *E. oleifera*, Interspecific, Intraspecific, Comparative mapping

## Abstract

**Background:**

Oil palm is an important perennial oil crop with an extremely long selection cycle of 10 to 12 years. As such, any tool that speeds up its genetic improvement process, such as marker-assisted breeding is invaluable. Previously, genetic linkage maps based on AFLP, RFLP and SSR markers were developed and QTLs for fatty acid composition and yield components identified. High density genetic maps of crosses of different genetic backgrounds are indispensable tools for investigating oil palm genetics. They are also useful for comparative mapping analyses to identify markers closely linked to traits of interest.

**Results:**

A 4.5 K customized oil palm SNP array was developed using the Illumina Infinium platform. The SNPs and 252 SSRs were genotyped on two mapping populations, an intraspecific cross with 87 palms and an interspecific cross with 108 palms. Parental maps with 16 linkage groups (LGs), were constructed for the three fruit forms of *E. guineensis* (*dura*, *pisifera* and *tenera*). Map resolution was further increased by integrating the *dura* and *pisifera* maps into an intraspecific integrated map with 1,331 markers spanning 1,867 cM. We also report the first map of a Colombian *E. oleifera*, comprising 10 LGs with 65 markers spanning 471 cM. Although not very dense due to the high level of homozygosity in *E. oleifera*, the LGs were successfully integrated with the LGs of the *tenera* map. Direct comparison between the parental maps identified 603 transferable markers polymorphic in at least two of the parents. Further analysis revealed a high degree of marker transferability covering 1,075 cM, between the intra- and interspecific integrated maps. The interspecific cross displayed higher segregation distortion than the intraspecific cross. However, inclusion of distorted markers in the genetic maps did not disrupt the marker order and no map expansion was observed.

**Conclusions:**

The high density SNP and SSR-based genetic maps reported in this paper have greatly improved marker density and genome coverage in comparison with the first reference map based on AFLP and SSR markers. Therefore, it is foreseen that they will be more useful for fine mapping of QTLs and whole genome association mapping studies in oil palm.

**Electronic supplementary material:**

The online version of this article (doi:10.1186/1471-2164-15-309) contains supplementary material, which is available to authorized users.

## Background

The oil palm is a diploid (2*n* = 2*x* = 32), outbreeding monocot with a genome of approximately 1.8 billion base-pairs [1]. The genus *Elaeis* consists of two species, *Elaeis guineensis* from Africa and *E. oleifera* from South America. *E. guineensis* has three fruit forms, *dura*, *pisifera* and *tenera. Tenera*, a hybrid of the two other fruit forms, is mainly grown in commercial plantations because of its higher oil yield compared to the *dura*, *E. oleifera* and interspecific hybrids of *E. oleifera* and *E. guineensis*. The *pisifera* palm is generally female sterile and exhibits fruit bunch abortion.

The Malaysian national average palm oil yield (OY) has stagnated at ~ 3.9 t/ha/yr for the past 20 years. The stagnation is particularly challenging to the Malaysian palm oil industry in its efforts to improve productivity. Land is scarce and there is no more than an additional 1.3 million ha for oil palm cultivation [2]. One important approach to bypass this constraint is to breed for higher yields. It has been postulated that oil palm can produce OY up to 18.2 t/ha/yr [3]. In fact, impressive progress has already been made in this direction with calculated OY as high as 12.2 t/h/yr [4] and 13.6 t/h/yr [5] in individual palms and experimental plots. This demonstrates the genetic potential of oil palm. Also the fatty acid composition (FAC) of palm oil has received a great deal of interest. About half of the palm oil is composed of saturated oil with 44.3% palmitic acid (C16:0) and 4.6% stearic acid (C18:0) [6]. As more unsaturated palm oil is desirable in some instances, one idea is to increase the proportion of oleic (C18:1) and linoleic (C18:2) acids.

For molecular breeding of oil palm, a number of QTLs associated with yield traits and FAC have previously been identified using restriction fragment length polymorphism (RFLP), amplified fragment length polymorphism (AFLP) and simple sequence repeat (SSR) markers [7–9]. However, marker density of the genetic maps remained low with large gaps between the markers and QTLs, leading to limited application of markers in breeding. Many more markers can now easily be identified using single nucleotide polymorphisms (SNPs) as already demonstrated in other plants. In apple (*Malus pumila* Mill.), marker density was dramatically improved from 1 per 3.8 cM to 1 per 0.5 cM by mapping 2,272 SNP markers [10]. The average map distance between markers for chickpea (*Cicer arietinum* L.) was also improved to 1 every 1.7 cM by inclusion of 697 SNP markers compared to previous reports [11]. There were also efforts to improve marker density by integrating maps from different crosses using SNP markers as bridges between maps, as in grape [12], potato [13], oilseed rape [14] and peach [15]. In oil palm, SNP markers are promising as they occur at relatively high frequency. Riju *et al*. found 16.8 – 17.5 SNPs/kbp in oil palm ESTs [16]. Therefore, SNPs can be used to improve genetic maps.

The present study was undertaken to improve the marker density of genetic maps from two separate mapping populations namely an *E. oleifera* × *E. guineensis* (*tenera*) interspecific cross and a *dura* × *pisifera* intraspecific cross which segregate for FAC and for yield components, respectively [9, 17]. The SNP markers developed to help with this were derived from oil palm genomic sequences [18]. A small number of these had previously been sucessfully tested using cleaved amplified polymorphic sequences (CAPS) and Illumina GoldenGate^®^ assays [19, 20]. Along with development of a number of automated genotyping platforms, it is now possible to assay a large set of SNP markers in several mapping populations simultaneously.

In recent years, many publications in potato [13], oilseeds [14], peach [15], tomato [21] and soybean [22] indicated the usefulness and reliability of the Illumina Infinium high throughput genotyping platform. In this study, an Infinium array of 4,451 oil palm customized SNPs, namely, OPSNP3, was used to develop saturated maps. These SNPs were selected based on their unique positions and wide-distribution on the published *pisifera* genome scaffolds generated from the whole genome sequencing project [1]. The high density genetic maps for the two crosses presented here can therefore improve the likelihood of locating markers more tightly linked to FAC and yield components and to ensure complete and even genome coverage for such analyses. At the same time, the study also cross-mapped a number of SSR-anchor markers from other published maps [8, 23, 24] to facilitate a comparison of maps developed from different genetic backgrounds.

Comparative mapping based on model plants or among closely related species has been reported for many plants, such as *Phaseolus vulgaris* L. and *Glycine max*[25, 26], *Populus alba* and *P. nigra*[27], *Pinus lambertiana* Dougl. and *Pinus taeda* L. [28], *Eucalyptus grandis*, *E. urophylla* and *E. globulus*[29] using various marker types. Different levels of conserved synteny were detected across the species, depending on their taxonomic distance. Comparative mapping is a very reliable method to establish the orthology of genomic regions in other species [30, 31] which can lead to gene discovery [31–34]. The extent of genome similarity and diversification between the intra- and interspecific hybrids of oil palm is not well documented. Mapping a common set of SNP and SSR markers onto their genetic maps facilitates direct comparison between the two genomes and makes it possible to establish the similarity between them. Homologous markers identified across the two hybrid maps can also be useful for further fine mapping, particularly for regions associated with traits of interest.

## Methods

### Mapping populations

**Figure 1:**
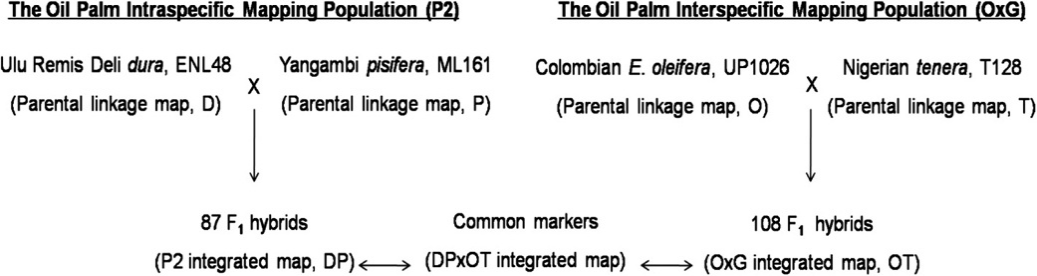
**Mapping populations.** The mapping strategy was to first construct a genetic linkage map for each parental palm in the P2 intraspecific cross *dura* (ENL48) x *pisifera* (ML161) and interspecific cross of *E. oleifera* x *E. guineensis* (OxG). A bi-parental integrated map was also constructed for P2 (labeled DP) and OxG (labeled OT). Subsequently, the DPxOT (/T) integrated map was constructed to show the common markers across the two populations.

Two mapping populations were obtained from different breeding backgrounds hereafter referred to as P2 and OxG. The family information and mapping strategies applied are illustrated in Figure 1. The P2 population is an intraspecific cross between Deli *dura* and Yangambi *pisifera*, a high yielding cross [35] from Federal Land Development Authority Malaysia (FELDA) which uses both parents extensively in its breeding programs. Preliminary parental maps containing AFLP, RFLP and SSR markers have been reported [17, 36]. The Yangambi *pisifera* parent has also been crossed with other Deli *duras* to develop an AFLP and SSR-based genetic linkage map [24]. The OxG population is an interspecific cross of a Colombian *E. oleifera* and a Nigerian *tenera*, obtained from United Plantations (UP) Berhad. A preliminary linkage map with AFLP, RFLP and a small number of SSR markers was previously constructed for the *tenera* parent. Markers broadly linked to QTLs associated with FAC were reported for OxG [9].

### Oil palm DNA extraction

Genomic DNA was extracted from frozen spear leaves (stored at -80°C) of palms using the modified CTAB method [37]. The DNA concentration and purity were measured using the NanoDrop spectrophotometer (NanoDrop Technologies Inc., Wilmington, DE) and visualized by 1% agarose gel electrophoresis. For SSR genotyping, DNA was diluted to 50 ng/ul. However, for the high throughput SNP assay using the Illumina platform, the DNA was first quantified using the Quant-iT™ PicoGreen^®^ dsDNA Reagent (Invitrogen, Carlsbad, CA) and subsequently normalized to 50 ng/ul.

### SNP assay

The SNP markers were derived from a collection of approximately 400,000 genomic sequences obtained from sequencing the hypomethylated regions of 8 palms using *GeneThresher*™ technology [18]. The criteria used to identify SNPs were – (1) the consensus sequence contained 40 – 60% GC nucleotides, (2) putative SNPs in either allele were supported by at least 2 reads, (3) to have at least 100 bases with no other SNPs within the 25-base window on the left and right of the target SNP to ensure that a 50-mer specific probe could be designed within the flanking region and (4) SNPs in repetitive or duplicated regions were removed. A small subset of SNPs produced were tested using CAPS assay [19, 38] to determine efficiency of the markers. Further filtering was done by mapping the sequences containing SNPs to the *E. guineensis* genome scaffolds [1]. SNP markers that mapped uniquely to the assembled genome were considered for further analysis. Of these, approximately 5,000 SNPs that were evenly distributed across the assembled genome were selected. A small panel of 96 SNPs (randomly chosen) were then validated on several mapping populations and a small set of samples from MPOB’s germplasm collections using the Illumina GoldenGate^®^ assay [20]. Upon validation of the genotyping efficiency, a final set of 4,451 SNPs, with a minimum Illumina design score of 0.9 were selected for developing the oil palm specific SNP array - OPSNP3.

The probes were printed on the 24-sample Infinium HD iSelect BeadChips platform (Illumina Inc., San Diego, CA). The sequence information for the SNP markers is available at http://genomsawit.mpob.gov.my with the nomenclature of SNPM (SNP marker) + a five digit number. The OPSNP3 array was used to genotype the P2 and OxG mapping populations using the Illumina Infinium II BeadChip platform. The SNP hybridization assay was carried out following the Illumina protocol (Illumina Inc., San Diego, CA). The DNA (200 ng) was isothermally amplified and incubated overnight followed by an enzymatic fragmentation. The fragments were purified before hybridizing onto the probes. After hybridization, allelic specificity was conferred by single base extension and fluorescent staining (green and red), with the signals detected by the Illumina BeadArray Scanner.

### SSR genotyping assay

In this study, additional oil palm SSR markers were generated using the M13-tailed primers (5’CACGACGTTGTAAAACGAC3’) as described by Ting *et al*. [17]. A separate M13 dye labelled primer was synthesized (forward primer-M13 and HEX-/ 6-FAM-/ NED-M13). The genotyping assay was carried out using the ABI3100 genetic analyzer (Applied Biosystems, Warrington, UK).

### SNP and SSR data analysis

Polymorphic SNPs were scored *aa*, *ab* or *bb* according to the default clustering algorithm of GenomeStudio^®^ Data Analysis Software (Illumina Inc., San Diego, CA). However, each genotype call was reassessed manually and rescored if necessary. SNPs that did not amplify or form well-separated clusters were excluded. Polymorphic SSRs were scored based on the observed segregation profiles (no.1, 5, 8 and 9) as reported previously [17]. In profile 1, heterozygous and homozygous genotypes for a segregating allele were scored as *ab* and *aa*. In profile 5, genotypes of two co-segregating alleles were scored as *aa* (homozygous for the first allele), *ab* (heterozygous) and *bb* (homozygous for the second allele). In profile 8, genotypes of three co-segregating alleles were scored as *aa* (homozygous for the common allele in both parents), *ab* (similar to the first parental genotype), *ac* (similar to the second parental genotype) and *bc* (heterozygote for the two different alleles in both parents). In profile 9, four co-segregating alleles were labeled as *a* and *b* for the two alleles in the first parent and *c* and *d* in the second parent. The observed genotypes in the hybrids were scored as *ac*, *bc*, *ad* and *bd* based on the combinations of the alleles. The data was incorporated into the existing data sets of P2 and OxG.

### Construction of the high density linkage maps

Linkage map analysis was performed separately for P2 and OxG using JoinMap^®^ 4.1 [39]. Both data sets were coded according to the format for cross-pollinator (CP). Chi-square analysis (χ^2^ test) was carried out to identify markers showing high levels of segregation distortion. Those with distorted ratios at p < 0.0001, or with ≥ 10% missing data (call rates < 90%), were excluded according to the criteria reported by Singh *et al*. [9].

The current software allows the construction of the two parental maps and the integrated map simultaneously using the maximum likelihood (ML) method [40]. The process of grouping markers and finding their best possible position was carried out as previously described [17]. Essentially the steps taken were as follow: (1) Linkage groups (LGs) were formed at a recombination frequency (RF) threshold of 0.2, (2) Haldane mapping function was used to transform the RF between markers into a map distance in centiMorgan (cM), (3) markers with a nearest neighbor stress (N.N. Stress) value > 4 cM were discarded from each LG (this would be expected to eliminate markers with unlikely genotypes, such as double recombination events in short genetic distances) and, (4) stability of the marker order on each LG was confirmed by altering the markers and comparing the results using MapChart 2.2 [41]. All other parameters applied in building the maps and ordering the markers used the default settings.

Integration of the cross-population P2 and OxG maps was carried out based on common markers using an extension to multiple populations of the method used for ordering markers in a single population [40]. Map distance was calculated using multipoint maximum likelihood via GenStat 15 [42]. The P2, OxG and integrated maps were aligned and compared using MapChart 2.2.

### Segregation distortion

Chi-square analysis (χ^2^ test) was carried out for each mapped marker (except for markers with segregation profiles 5, 8 and 9). The segregation ratios of the maternal palm (for markers with segregation profile 1 < *ab*x*aa* >) and the paternal palm (for markers with segregation profile 1 < *aa*x*ab* > were determined by calculating the proportion of alleles obtained from the respective first grandparents. The assignment ‘first grandparent’ versus ‘second grandparent’ depended on the phasing of markers (JoinMap^®^4.1 [39], when the LGs were formed). For a marker with segregation type < *ab*x*aa* > and phase {0-} ({1-}), the individual scored *aa* inherits the maternal allele from the maternal first (second) grandparent, whereas for an individual scored *ab* the maternal allele is inherited from the maternal second (first) grandparent. Hereafter, the ‘first grandparent’ will be referred to as grandmother. In order to get a better understanding of the genomic regions involved in segregation distortion, the proportion of alleles inherited from the respective grandmothers were estimated for both populations and plotted against the positions of markers on the linkage map.

## Results

### Oil palm SNP (OPSNP3) array

A total of 4,451 customized oil palm SNPs were genotyped on the P2 intraspecific and OxG interspecific mapping populations using the Illumina Infinium platform. The proportion of single base changes observed was 61% (2,708) transitions (1,333 A/G and 1,375 T/C) and 39% (1,743) transversions (477 A/C, 462 T/G, 470 A/T, 334 C/G). The number of transition SNPs were ~ 1.5 times the number of transversions, close to the values found in chickpea [11], melon [43] and cacao [44].

### Assessment of informative markers

The SNP genotyping results showed very high call rates which ranged from 97 to 98% for P2 and 91 to 97% for OxG, indicating the efficiency of the genotyping platform used. The majority of the SNPs were clustered in clearly defined profiles with only a small number (37 in P2 and 58 in OxG) displaying insufficient separation of the allele clusters. These SNPs were excluded from further analysis. A total of 3,082 and 3,299 of the SNPs were monomorphic in P2 and OxG, respectively; they were also excluded from linkage analysis in the bi-parental populations. The P2 intraspecific hybrid revealed more informative SNPs (30%) than the OxG interspecific hybrid (24%).

In P2, a total of 1,324 informative SNPs and an additional 53 SSRs were identified and incorporated into the existing data set (342 SSRs) previously described [17]. The final data set used for linkage analysis is summarized in Table 1. Of the 1,324 SNPs, 1,109 segregated in two clusters (*aa* and *ab*) corresponding to either homozygotes or heterozygotes, and 215 co-segregated in three clusters (*aa*, *ab* and *bb*). In the SSR analysis, profile 1 (299 SSRs), profile 5 (21 SSRs), profile 8 (53 SSRs) and profile 9 (22 SSRs) were observed as previously reported [17]. Polymorphism was higher in the male parent (*pisifera*), in agreement with previous reports [17]. In OxG, 1,065 polymorphic SNPs and 316 SSRs (including 199 new SSRs) were identified with the majority segregating from the *tenera* parent. Across these markers, the heterozygosity level for the male parental palm was 0.9. On the contrary, *E. oleifera* showed low levels of heterozygosity (0.1) with only 148 polymorphic markers (68 SNPs and 80 SSRs). This also help to explain why only one SNP was polymorphic in both parents of OxG.

**Table 1:** **Summary of SSR and SNP markers in P2 and OxG mapping populations**

	P2			OxG		
	SNP	SSR	Sub-total	SNP	SSR	Sub-total
Numbers of polymorphic alleles:						
1 (profile 1)	1,109	299	1,408	1,064	290	1,354
2 (profile 5)	215	21	236	1	0	1
3 (profile 8)	-	53	53	0	6	6
4 (profile 9)	-	22	22	0	20	20
Sub-total	1,324	395	1,719	1,065	316	1,381
Segregation distortion:						
p < 0.1	158	60	218	375	87	462
p < 0.05	104	37	141	243	68	311
p < 0.01	10	6	16	120	40	160
p < 0.005	3	4	7	93	31	124
p < 0.001	-	-	-	23	9	32
p < 0.0005	-	-	-	5	3	8
Ungrouped	38	26	64	29	22	51
Unmapped	76	238	314	16	140	156
Mapped	1,210	131	1,341	1,020	154	1,174

In P2, 158 SNPs (12%) and 60 SSRs (15%) exhibited segregation distortion at p < 0.1, including p < 0.05 (104 SNPs and 37 SSRs), p < 0.01 (ten SNPs and six SSRs) and p < 0.005 (three SNPs and four SSRs) (Table 1). However, most of the markers followed the expected segregation ratios. In OxG, approximately 65% of the SNPs and 73% of the SSRs agreed with the expected transmission ratios. The remainder (375 SNPs and 87 SSRs) demonstrated various degrees of distortion at – p < 0.1, including p < 0.05 (243 SNPs and 68 SSRs), p < 0.01 (120 SNPs and 40 SSRs), p < 0.005 (93 SNPs and 31 SSRs), p < 0.001 (23 SNPs and nine SSRs) and p < 0.0005 (five SNPs and three SSRs).

### P2 parental maps and intraspecific integrated map

A total of 1,324 SNP and 395 SSR markers were analyzed for construction of the genetic map in the P2 mapping population. Of these, 1,210 SNPs and 131 SSRs (Table 1) were assembled into the 16 linkage groups (LGs) of the *dura* (D) and *pisifera* (P) parental maps (Additional file 1). The number of markers mapped in each LG ranged from 11 to 70 in the D, and 15 to 125 in the P map. The lengths of LG varied from 50 (D16) to 144 cM (D8) and 61 (P5) to 215 cM (P4), respectively. The marker content and length of each LG are shown in more detail in Additional file 2. The resulting P2 parental maps covered 1,469 and 1,917 cM with 622 and 921 markers for D and P, respectively.

In P2, 213 codominant markers (188 SNPs and 25 SSRs) were identified for all 16 LGs. These markers provided multiple anchors for integrating the two parental maps into a high density intraspecific integrated genetic map (labeled DP). The common markers were unevenly distributed among the LGs, with DP1 possessing 31 and DP5 only two markers. Other LGs contained different numbers of the common markers, ranging from 20 to 25 (in three LGs), 15 – 19 (three LGs), 10 – 14 (five LGs) and 5 – 9 (four LGs). The marker order in the DP integrated map was highly consistent with the individual D and P maps (Additional file 1). The map density increased to 1,331 markers/1,867 cM with an average distance between markers of 1.4 cM, reducing the average gap compared to that in D (2.4 cM) and P (2.1 cM). However, there were still low density regions with intervals ranging from 11 to 18 cM in LGs DP1, 3, 4, 5, 9, 11, 13, 14 and 15 (Additional file 2).

### OxG parental maps and a partial interspecific integrated map

A total of 1,381 polymorphic markers - 1,065 SNPs and 316 SSRs - were scored in the OxG mapping population and used for linkage analysis. Of these, 51 were ungrouped and 156 were removed due to high stress values (≥ 4 cM). The remaining 1,174 (1,020 SNPs and 154 SSRs) were mapped in the *tenera* (T) and *E. oleifera* (O) parental maps. The T map had 16 LGs with T4 the longest group (217 cM) with 140 markers. On the other hand, LG T5 was among the smallest groups with only 24 markers spanning 61 cM. By comparison, T is a remarkably high density map (1,121 markers/1,759 cM) compared with O, which has only 10 sparse LGs with 65 markers/471 cM. Although not very dense, the 10 LGs were successfully integrated with LGs from the T map. The integrated LGs were labeled OT1, 4, 6, 7, 8, 10, 11, 12, 13 and 15. As the majority of markers were from *tenera*, the integrated LGs are very similar to the T map (Additional file 1).

### Genome-wide map comparison between P2 and OxG

A total of 603 transferable markers (550 SNPs and 53 SSRs) were co-mapped in at least two of the D, P, O and T parental maps. Assembly of these shortlisted markers in a genetic map labeled DPxOT (/T) indicates the conserved level of homology between the two crosses. The use of a subset of markers explains why the density of cross-species DPxOT (/T) map is less than the bi-parental integrated maps (DP and OT). Alignment of each LG in DPxOT (/T) with LG of D, P and DP (P2) and LG of O, T and OT (OxG) is presented in Additional file 1. Comparison of the LGs is also shown via Circos plots (see Additional file 3).

Comparing the two hybrid maps, a total of 450 transferable markers were detected across the P2 and OxG populations covering a total map length of approximately 1,075 cM. The transferable markers were well distributed in LGs 3 and 16. However, many other markers were distributed unevenly along the LGs and fragmented into smaller blocks of common markers. The pattern was observed particularly in LGs 9, 11 and 15 where two distinct blocks of transferable markers were located at opposite arms of the compared groups. Loci that mapped in between the blocks were unique SNPs and SSRs polymorphic in either P2 or OxG.

### Segregation distortion

**Figure 2:**
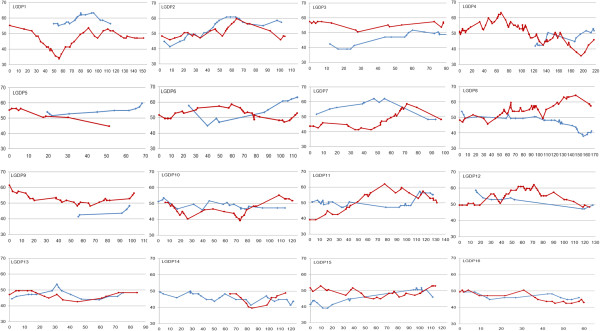
**Distribution of segregation distortion across the 16 integrated linkage groups of P2** **(DP)** **.** Y-axis represents proportion (%) of grandmaternal alleles in ENL48 (blue line) and in ML161 (red line) whereas, X-axis represents map position (cM).

**Figure 3:**
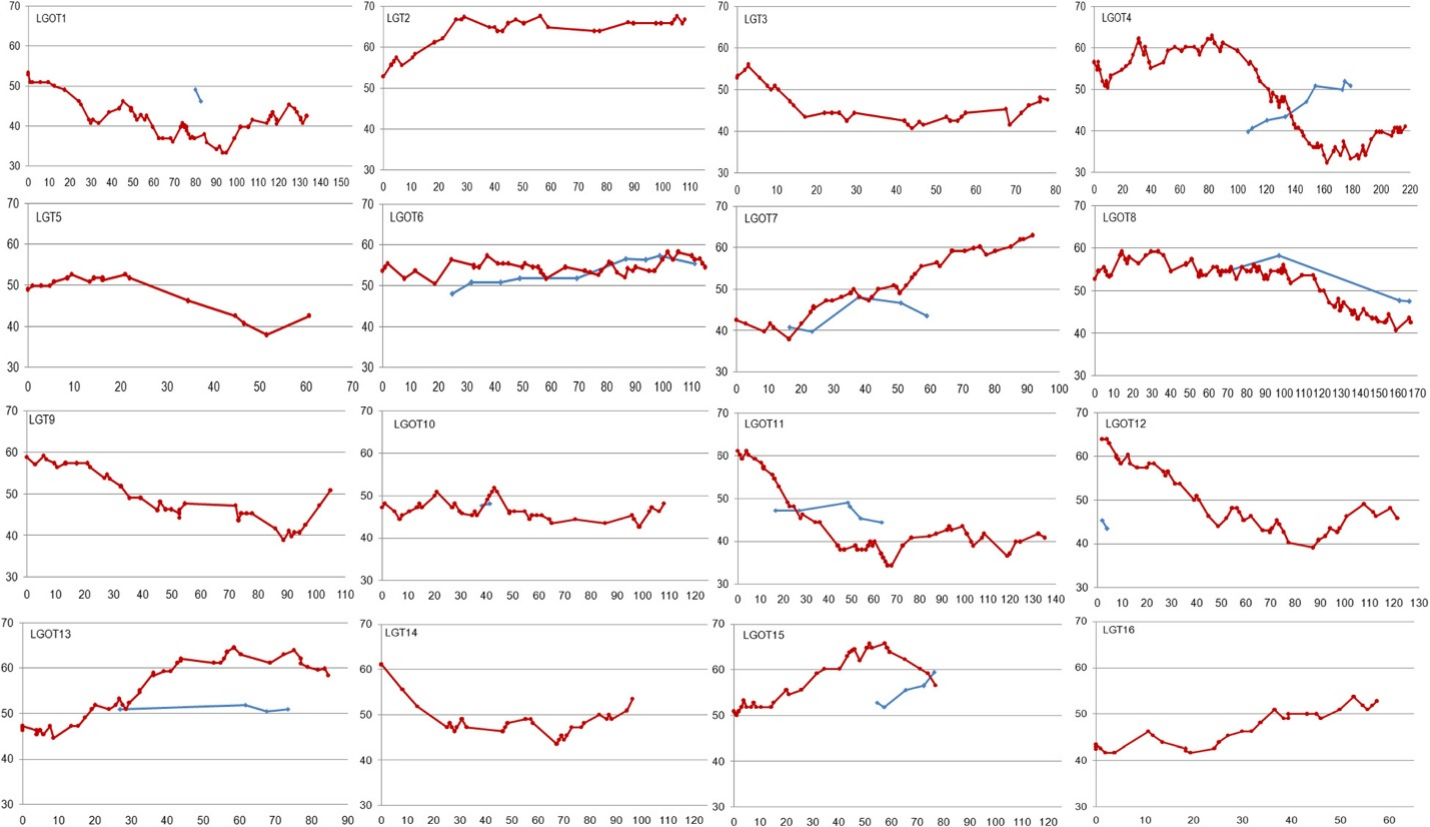
**Distribution of segregation distortion across the 10 integrated linkage groups** **(** **OT** **)** **and additional six LGs T of OxG.** Y-axis represents proportion (%) of grandmaternal alleles in UP1026 (blue line) and in T128 (red line) whereas, X-axis represents map position (cM).

Unequal segregation distortion was observed in the P2 intraspecific and OxG interspecific hybrids. In OxG, 35% (363 SNPs and 49 SSRs) of the mapped markers showed significant deviation from the expected Mendelian segregation ratios. The distorted SNPs showed no preference for transition or transversion, as the transition/transversion ratio remained close to 1.7. The segregation distortion observed for SSR markers involved 35 di-, six tri-, three mono-, two tetra-, two compound- and one hexa-nucleotide repeat unit motifs. The distorted SNP and SSR markers were distributed non-randomly among the LGs with more distorted markers (40 – 81%) in LGs OT1, T2, OT4, OT7, OT11, OT13 and OT15. Other LGs showed a lower proportion of distorted markers (1 – 23%). About 50% of the deviations in the OxG cross were in favor of the *tenera* parent and the other half biased towards *E. oleifera*. In contrast, only a few skewed markers (159) were observed in P2. Distorted markers which ranged from 2 to 23% were observed in 14 LGs, with two LGs (DP13 and 16) free from any distorted marker.The two LGs in P2 (DP13 and 16) that were free from distorted markers had for both parents a proportion of grandmaternal alleles ranging between 43 – 54% (Figure 2). However, for almost all other LGs, many regions deviated considerably from 50% with values going up to 63% and down to 33%. Similarly in OxG, LG OT6 showed very little segregation distortion with values between 48 – 58% for both parents (Figure 3). In other genomic regions the segregation distortion was considerable with values as high as 68% and as low as 33%. It has also been observed that markers with segregation ratios differing strongly from 50% clustered together in specific genomic regions; these may contain genes involved in gamete selection.

## Discussion

The rapid development of high throughput genotyping techniques makes them very cost effective for assaying large numbers of markers, which is useful in genetic mapping. In this study, the generation of SNP and SSR marker data was greatly accelerated (compared with conventional gel-based methods) using high throughput genotyping platforms. The Illumina Infinium customized OPSNP3 genotyping array and ABI3100 capillary-based fragment analysis allowed us to genotype the same set of SNP and SSR markers in two mapping populations simultaneously and data generation was completed within a short time. Genotyping of 252 SSR and 4,451 SNP markers across 199 palms (including parental palms) of P2 and OxG took less than three months. Furthermore both systems detect co-dominant alleles with differences as small as one base thus minimizing errors in scoring the genotype of markers. The high quality marker data helped in generation of marker linkage groups without obvious inflation of map distances that can be caused by false linkages [45]. The genotyping cost was considered reasonable for the generation of data from a large set of samples and markers.

The genetic maps constructed in this study have thus successfully extended the coverage of the previous *tenera* map [9] and the *dura* and *pisifera* maps [17]. A large set of SNP and SSR markers was used to replace the existing AFLP and RFLP markers to simplify the process of marker ordering in the genetic maps. The average gap between markers in the new maps (estimated as map length over the number of mapped markers) was greatly reduced from one marker every 7.2 cM to one marker every 1.6 cM in *tenera* (T), 5.4 cM to 2.4 cM in *dura* (D) and 5.5 cM to 2.1 cM in *pisifera* (P) maps. The gap was down to 1.4 cM in the DP intraspecific integrated genetic map from the previous 1.8 cM [23] for an integrated *tenera* x *dura* map. To the best of our knowledge, the parental and integrated maps presented in this study are the most comprehensive genetic linkage maps published for oil palm to date.

For the OxG interspecific cross, a previous effort failed to construct a map for the female parent, *E. oleifera*, mainly due to the limited number of AFLP and RFLP markers used [9]. In this study, 10 small LGs with a majority derived from SNP markers were constructed for the *E. oleifera* parent. Although a large number of SNP markers and a significant number of *E. oleifera*-derived SSR markers were used, we still could not detect sufficient polymorphisms to saturate the map. This strongly suggests that the Colombian *E. oleifera* (used in creating the OxG hybrid) is very homozygous. Based upon this finding, future studies should include crossing Colombian *E. oleifera* with a distantly related *E. oleifera* in order to increase the level of heterozygosity in *E. oleifera*. Several rounds of such crossing with different *E. oleifera* may have to be done first, before generating the OxG hybrid. Nevertheless, this study has, for the first time, produced an *E. oleifera* map, albeit with only limited markers (65 markers/471 cM). Furthermore, the *E. oleifera* map was successfully integrated with the *tenera* parental map.

The established high density genetic maps proved useful for comparative mapping analyses. Previously, it was difficult to compare the published genetic maps of oil palm, as different labs used different markers. The markers used in this study also included the published SSRs thus enabling the current maps to be linked to the previously published maps [8, 9, 17, 23, 24]. Comparisons based on these anchoring markers is presented in Additional file 4. More importantly, SSR markers linked to QTLs for traits such as bunch number, bunch weight, fresh fruit bunch yield, fruit to bunch ratio, kernel to fruit ratio [8] and C16:0 and C18:1 [9], were successfully localized in the maps reported here. The mapping populations in this study also segregate for similar traits and the SSR markers will therefore be useful to indicate the LG locations of the QTLs and the nearby SNPs will help to reveal and validate similar QTL regions for yield components and FAC in P2 and OxG, respectively.

All genetic maps constructed in this study can be directly compared because of the high frequency of transferable markers. Approximately 75% of the mapped markers were cross-mapped in at least two parental maps of *dura*, *pisifera*, *E. oleifera* and *tenera*, and serve as good anchors in connecting the maps. The transferable markers will be good starting points to analyze other mapping populations and germplasm. The known map positions will be an added advantage, particularly for estimation of linkage disequilibrium (LD), assessment of genetic diversity and association mapping in oil palm. In oilseed rape for example, over 4,000 SNP markers derived from the genetic map were analyzed on different germplasm collections [14]. Combination of the map information and genotyping data generated from the germplasm analysis allowed the determination of genetic diversity and the extent of LD. From the results, the authors also identified a smaller set of markers suitable for genome-wide association studies. This avoids the use of markers with an uneven or narrow distribution which can severely affect the association of markers to traits of interest. Furthermore, inclusion of transferable markers from specific map locations (such as targeted QTL regions) is useful in identifying and validating marker (s) linked to similar traits in germplasm collections. For example, in wheat, SSR markers associated with QTLs for plant height, spike length, grain yield and other traits on chromosome 4A, were also associated with the traits in germplasm collections [46].

This study reports the first comparative mapping analysis between intra- and interspecific hybrids of oil palm. The consistency of marker order and the limited map expansion observed made comparative mapping of the P2 and OxG crosses relatively simple. A high level of congruence between the genetic maps was observed, revealing 57 – 61% transferable markers between the two hybrid genomes. The transferable markers identified in each LG can help in identifying the corresponding genomic regions in other fruit forms of oil palm, leading to further fine mapping and structural analyses. In *Brassica*, common QTLs for plant height, flowering time and seed traits were identified in *B. rapa*, *B. napus* and *B. juncea* through synteny analysis [34]. However, the study also examined the expression levels of genes between the species segregating for the QTLs and the counterparts that did not segregate for the same trait. This expression level information, when compared with the whole genome sequencing data of *Brassica* ssp and *A. thaliana* led to the discovery of several potentially useful candidate markers for explaining this variation. With the availability of the oil palm genome sequence [1], the anchor markers can be linked to the oil palm physical map. This will facilitate further saturation of the LGs where specific SSRs or SNPs can be designed in areas with large genetic gaps. In fact, the P2 genetic maps constructed in this study showed excellent co-linearity in marker order consistent with the oil palm genome sequence assemblies [1]. Comparison with the whole genome sequence will be very helpful in identifying markers more tightly linked to traits than the ones discovered so far.

Comparative mapping also revealed that the distorted markers mainly occurred in clusters, possibly in the segregation distortion regions (SDRs) [47–50]. However, there is no intimation that SDRs are found at the same map regions or involve the same groups of markers across P2 and OxG. Instead, the SDRs are largely unique to each population and parental palm reflecting the respective polymorphisms and recombination events as has also been observed in potato [13] and barley [48]. The SDRs also may indicate genomic regions involved in selection, e.g. of gametes in the maternal and paternal meioses. A greater tendency for segregation distortion has always been reported in interspecific, or wide crosses in plants [50–52]. Similar results were obtained in this study with the distortion rate higher in the OxG interspecific cross than in the P2 intraspecific cross. The higher segregation distortion in OxG is not surprising as it reflects the substantial genetic divergence and unequal levels of heterozygosity in the two parental palm species. The distortion segregation rate in P2 was similar to that observed in the *E. guineensis* intraspecific map [23].

In oil palm, SDRs have not been extensively studied compared to the situation in rice, wheat, maize and soybean where they are often associated with genes causing gametic competition [51], gametophytic selection (*ga*) [53–56] and sterility [54, 57, 58]. In maize, preferential fertilization has been frequently reported in pollen with the *ga1* allele that mediates pollen – pistil interactions [54]. This results in male gamete competition during transmission which might cause segregation distortion at the *ga* region. In rice, it was postulated that other chromosomal regions involved in pistil interaction might also interact with *ga* alleles and cause segregation distortion [52]. However, the results on SDRs in P2 and OxG indicate that in many regions of the genome, there is a preference for one of the grandparental alleles due to some form of selection (either gametic or zygotic). Regions with segregation ratios close to 30% or 70% may contain incompatibility genes. These regions are of interest as they may give insight into the selection and evolution of a species [59].

## Conclusions

This study established SNP and SSR high resolution genetic maps for two oil palm hybrids. Genome-wide comparisons between the maps identified transferable markers in the intra- and interspecific integrated maps. The high density genetic maps will be useful for further fine mapping, particularly in finding markers closely linked to QTL associated with traits of interest. The map information therefore can facilitate map-based cloning of the genes, especially through the use of the oil palm genome sequencing data. We foresee these maps being used extensively for QTL detection and whole genome association mapping in oil palm.

### Availability of supporting data

The sequence information for the SNP markers is available at http://genomsawit.mpob.gov.my.

## Electronic supplementary material

Additional file 1: **Alignment of oil palm genetic maps developed for intraspecific** **(** **P2** **)** **and interspecific** **(** **OxG** **)** **crosses using common markers.** The P2 bi-parental integrated (DP), *dura* parental (D) and *pisifera* parental (P) maps are shown on the left and OxG bi-parental integrated (OT), *E. oleifera* parental (O) and *tenera* parental (T) maps on the right. The integrated map of P2 and OxG is shown at the centre, labeled DPxOT/T. Haldane genetic distance (cM) is indicated by the ruler on the left of the map. Common markers that co-mapped across D, P, DP, DPxOT/T and T are indicated in red. Nomenclature for markers is: SNPM (SNP), mEgCIR (genomic SSR from [8, 23], sEg (*E. guineensis* EST-SSR), sMg (*E. guineensis* genomic-SSR), sMo (*E. oleifera* genomic-SSR), sPSc (SSR developed from *E. guineensis* scaffold data). Markers showing distorted segregation are marked by *representing significance, *viz*.,*p < 0.1,**p < 0.05,***p < 0.01,****p < 0.005,*****p < 0.001 and ******p < 0.0005. (ZIP 6 MB)

Additional file 2: **Information on P2 (D, P and DP) and OxG (O, T and OT) genetic maps as well as the cross-population integrated map (DPxOT/T).** (DOCX 62 KB)

Additional file 3: **A closer view of comparative linkage groups (LGs) of** ***dura*** **(D),** ***pisifera*** **(P),** ***E. oleifera*** **(O) and** ***tenera*** **(T) plotted with Circos [**[60]**].** LGs 1 – 16 are scaled in Haldane genetic distance (cM) and represented by different color coded lines. (DOCX 827 KB)

Additional file 4: **Comparative linkage groups (LGs) between the current maps and previously published oil palm maps.** The P2 integrated map (DP) and OxG integrated map (OT/T) are aligned with maps developed by Billotte *et al*. [8], Singh *et al*. [9], Ting *et al*. [17] and Seng *et al*. [24] based on common RFLP and SSR markers. (DOCX 664 KB)
