# Supplementary figures and images for: High density SNP and SSR-based genetic maps of two independent oil palm hybrids
Source: BMC Genomics. 2014 Apr 27;15(1):309. doi: 10.1186/1471-2164-15-309 (PMC4234488; doi:10.1186/1471-2164-15-309)

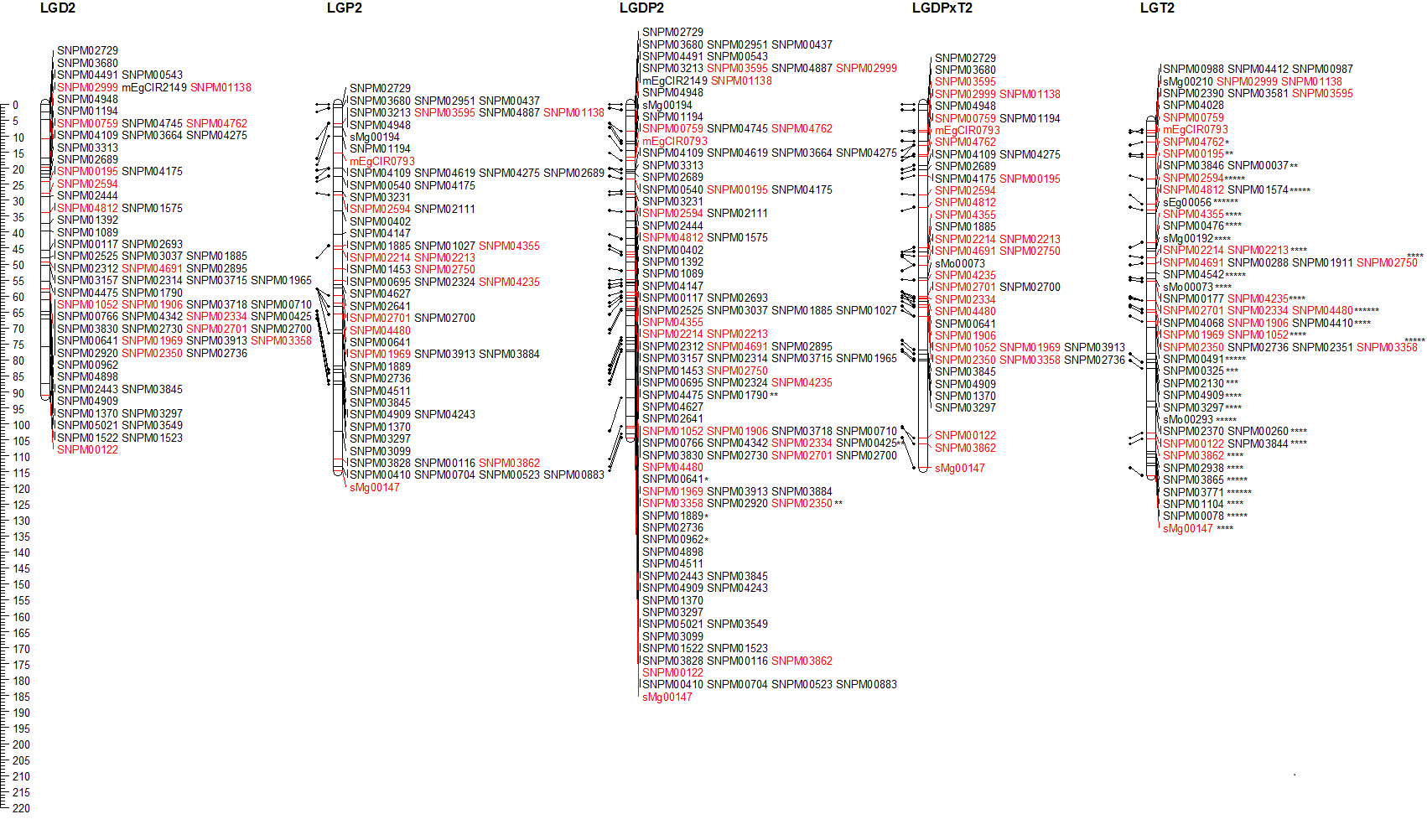

Supplement: Supplementary file 1 — Additional file 1: Alignment of oil palm genetic maps developed for intraspecific ( P2 ) and interspecific ( OxG ) crosses using common markers. The P2 bi-parental integrated (DP), dura parental (D) and pisifera parental (P) maps are shown on the left and OxG bi-parental integrated (OT), E. oleifera parental (O) and tenera parental (T) maps on the right. The integrated map of P2 and OxG is shown at the centre, labeled DPxOT/T. Haldane genetic distance (cM) is indicated by the ruler on the left of the map. Common markers that co-mapped across D, P, DP, DPxOT/T and T are indicated in red. Nomenclature for markers is: SNPM (SNP), mEgCIR (genomic SSR from [8, 23], sEg (E. guineensis EST-SSR), sMg (E. guineensis genomic-SSR), sMo (E. oleifera genomic-SSR), sPSc (SSR developed from E. guineensis scaffold data). Markers showing distorted segregation are marked by *representing significance, viz.,*p < 0.1,**p < 0.05,***p < 0.01,****p < 0.005,*****p < 0.001 and ******p < 0.0005. (ZIP 6 MB) [file 12864_2013_7049_MOESM1_ESM.zip › LG2.tif]

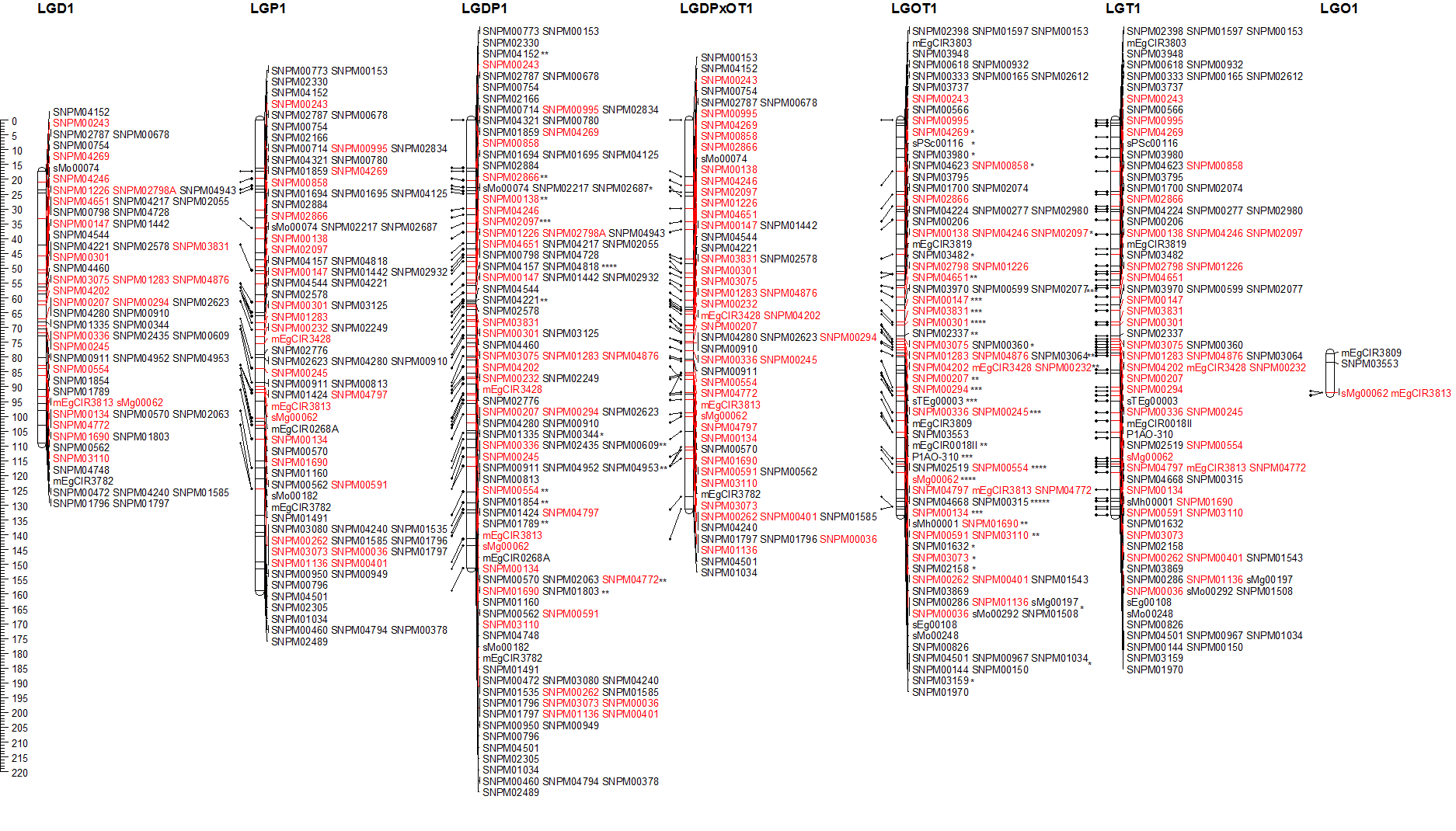

Supplement: Supplementary file 1 — Additional file 1: Alignment of oil palm genetic maps developed for intraspecific ( P2 ) and interspecific ( OxG ) crosses using common markers. The P2 bi-parental integrated (DP), dura parental (D) and pisifera parental (P) maps are shown on the left and OxG bi-parental integrated (OT), E. oleifera parental (O) and tenera parental (T) maps on the right. The integrated map of P2 and OxG is shown at the centre, labeled DPxOT/T. Haldane genetic distance (cM) is indicated by the ruler on the left of the map. Common markers that co-mapped across D, P, DP, DPxOT/T and T are indicated in red. Nomenclature for markers is: SNPM (SNP), mEgCIR (genomic SSR from [8, 23], sEg (E. guineensis EST-SSR), sMg (E. guineensis genomic-SSR), sMo (E. oleifera genomic-SSR), sPSc (SSR developed from E. guineensis scaffold data). Markers showing distorted segregation are marked by *representing significance, viz.,*p < 0.1,**p < 0.05,***p < 0.01,****p < 0.005,*****p < 0.001 and ******p < 0.0005. (ZIP 6 MB) [file 12864_2013_7049_MOESM1_ESM.zip › LG1.tif]

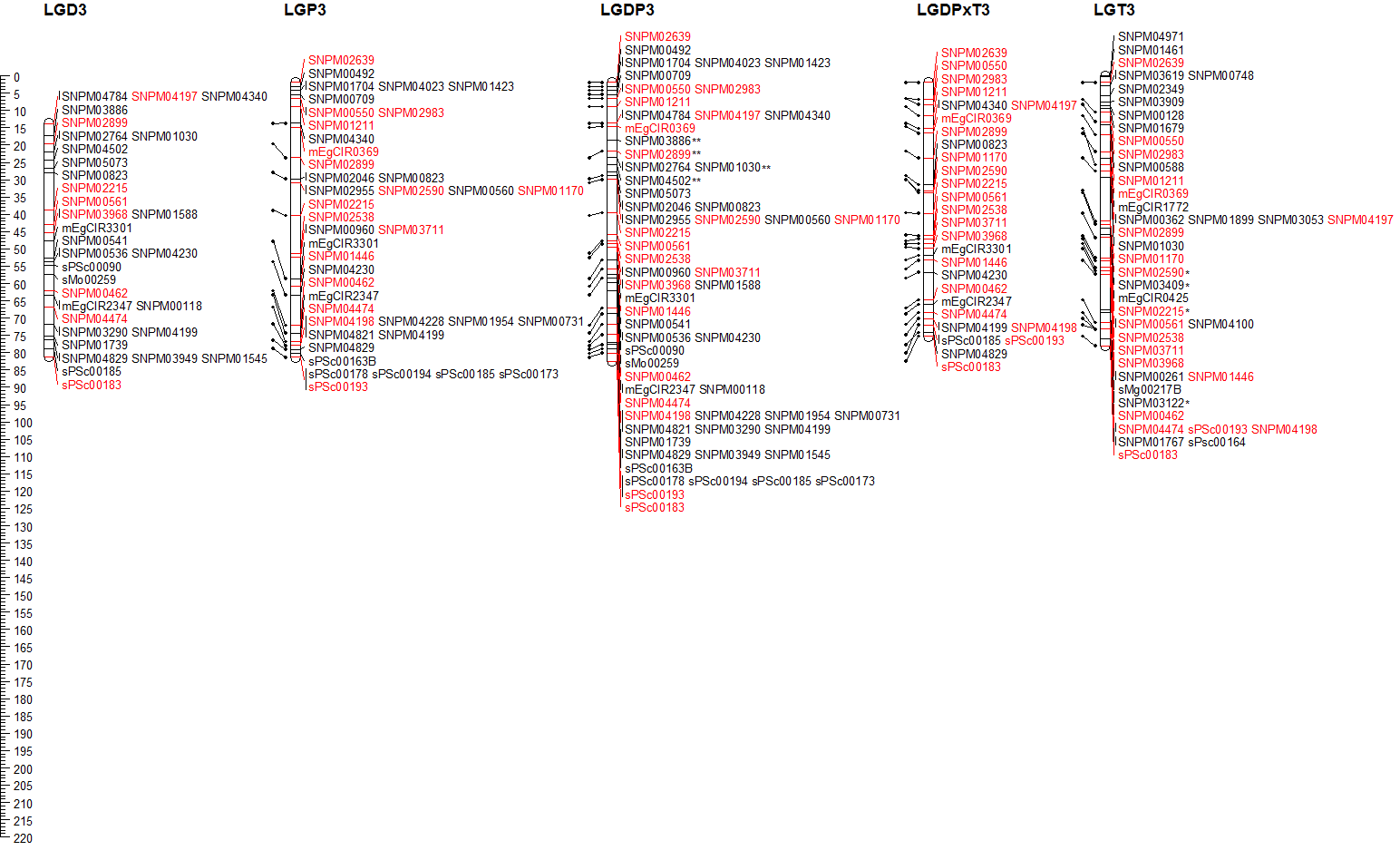

Supplement: Supplementary file 1 — Additional file 1: Alignment of oil palm genetic maps developed for intraspecific ( P2 ) and interspecific ( OxG ) crosses using common markers. The P2 bi-parental integrated (DP), dura parental (D) and pisifera parental (P) maps are shown on the left and OxG bi-parental integrated (OT), E. oleifera parental (O) and tenera parental (T) maps on the right. The integrated map of P2 and OxG is shown at the centre, labeled DPxOT/T. Haldane genetic distance (cM) is indicated by the ruler on the left of the map. Common markers that co-mapped across D, P, DP, DPxOT/T and T are indicated in red. Nomenclature for markers is: SNPM (SNP), mEgCIR (genomic SSR from [8, 23], sEg (E. guineensis EST-SSR), sMg (E. guineensis genomic-SSR), sMo (E. oleifera genomic-SSR), sPSc (SSR developed from E. guineensis scaffold data). Markers showing distorted segregation are marked by *representing significance, viz.,*p < 0.1,**p < 0.05,***p < 0.01,****p < 0.005,*****p < 0.001 and ******p < 0.0005. (ZIP 6 MB) [file 12864_2013_7049_MOESM1_ESM.zip › LG3.tif]

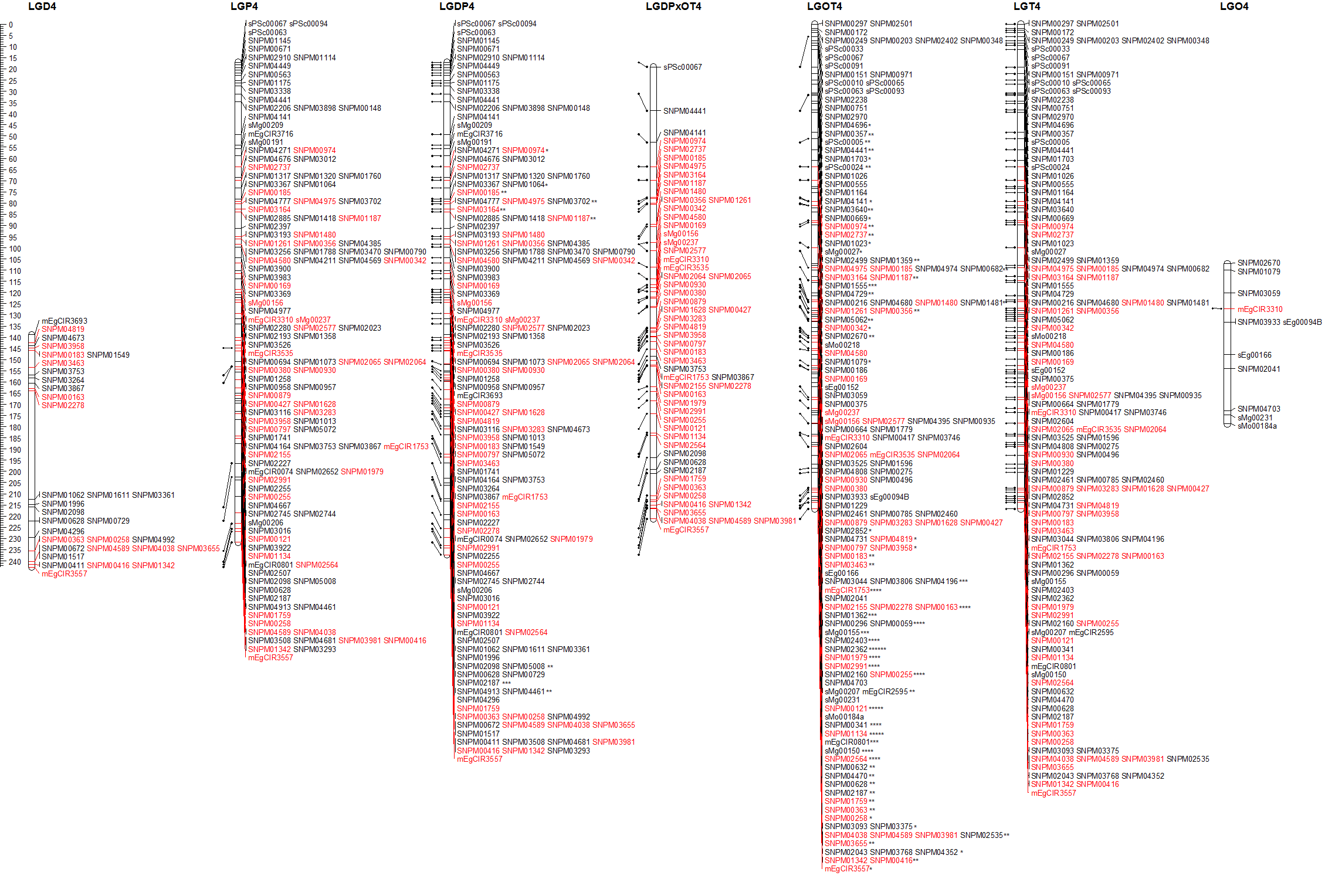

Supplement: Supplementary file 1 — Additional file 1: Alignment of oil palm genetic maps developed for intraspecific ( P2 ) and interspecific ( OxG ) crosses using common markers. The P2 bi-parental integrated (DP), dura parental (D) and pisifera parental (P) maps are shown on the left and OxG bi-parental integrated (OT), E. oleifera parental (O) and tenera parental (T) maps on the right. The integrated map of P2 and OxG is shown at the centre, labeled DPxOT/T. Haldane genetic distance (cM) is indicated by the ruler on the left of the map. Common markers that co-mapped across D, P, DP, DPxOT/T and T are indicated in red. Nomenclature for markers is: SNPM (SNP), mEgCIR (genomic SSR from [8, 23], sEg (E. guineensis EST-SSR), sMg (E. guineensis genomic-SSR), sMo (E. oleifera genomic-SSR), sPSc (SSR developed from E. guineensis scaffold data). Markers showing distorted segregation are marked by *representing significance, viz.,*p < 0.1,**p < 0.05,***p < 0.01,****p < 0.005,*****p < 0.001 and ******p < 0.0005. (ZIP 6 MB) [file 12864_2013_7049_MOESM1_ESM.zip › LG4.tif]

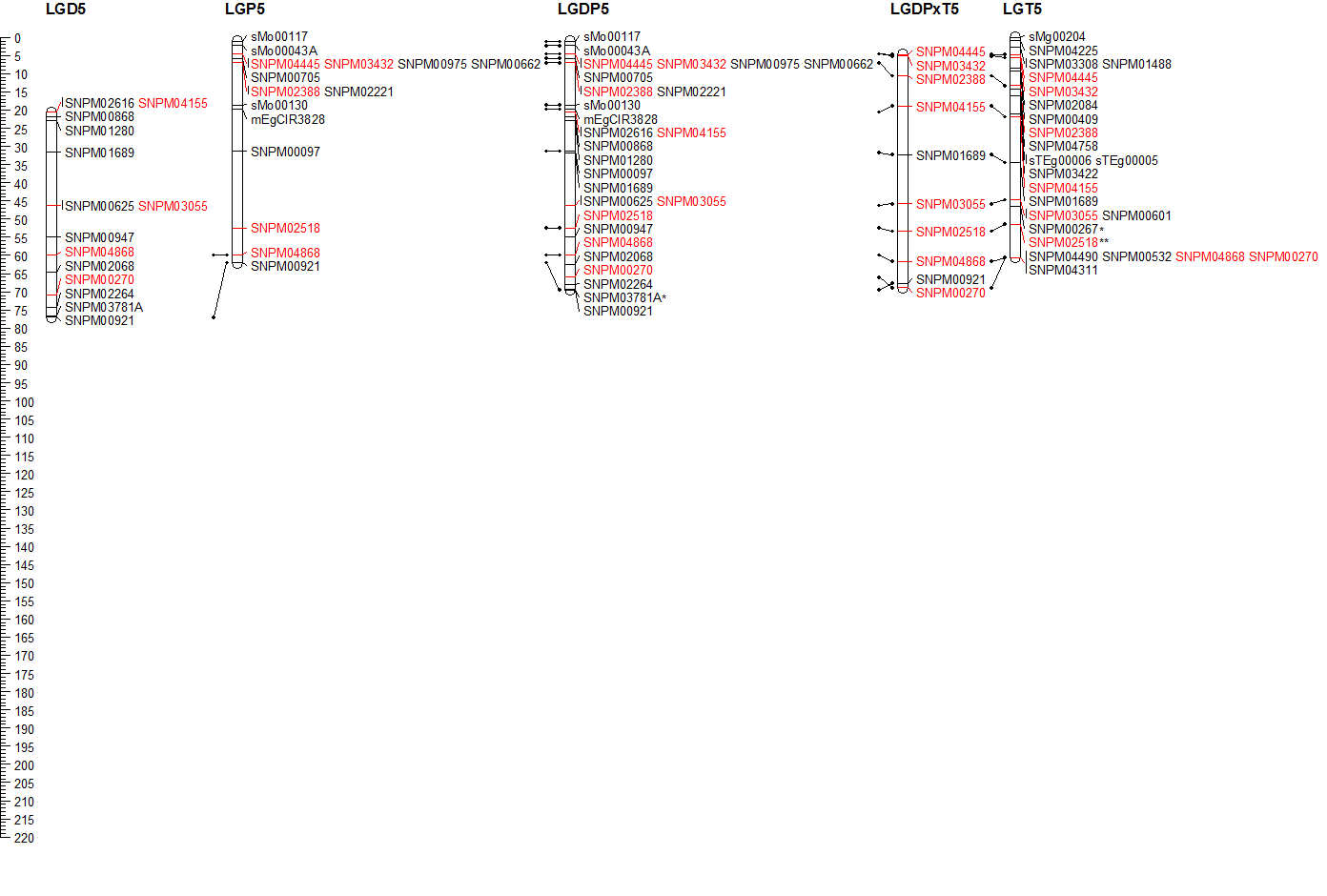

Supplement: Supplementary file 1 — Additional file 1: Alignment of oil palm genetic maps developed for intraspecific ( P2 ) and interspecific ( OxG ) crosses using common markers. The P2 bi-parental integrated (DP), dura parental (D) and pisifera parental (P) maps are shown on the left and OxG bi-parental integrated (OT), E. oleifera parental (O) and tenera parental (T) maps on the right. The integrated map of P2 and OxG is shown at the centre, labeled DPxOT/T. Haldane genetic distance (cM) is indicated by the ruler on the left of the map. Common markers that co-mapped across D, P, DP, DPxOT/T and T are indicated in red. Nomenclature for markers is: SNPM (SNP), mEgCIR (genomic SSR from [8, 23], sEg (E. guineensis EST-SSR), sMg (E. guineensis genomic-SSR), sMo (E. oleifera genomic-SSR), sPSc (SSR developed from E. guineensis scaffold data). Markers showing distorted segregation are marked by *representing significance, viz.,*p < 0.1,**p < 0.05,***p < 0.01,****p < 0.005,*****p < 0.001 and ******p < 0.0005. (ZIP 6 MB) [file 12864_2013_7049_MOESM1_ESM.zip › LG5.tif]

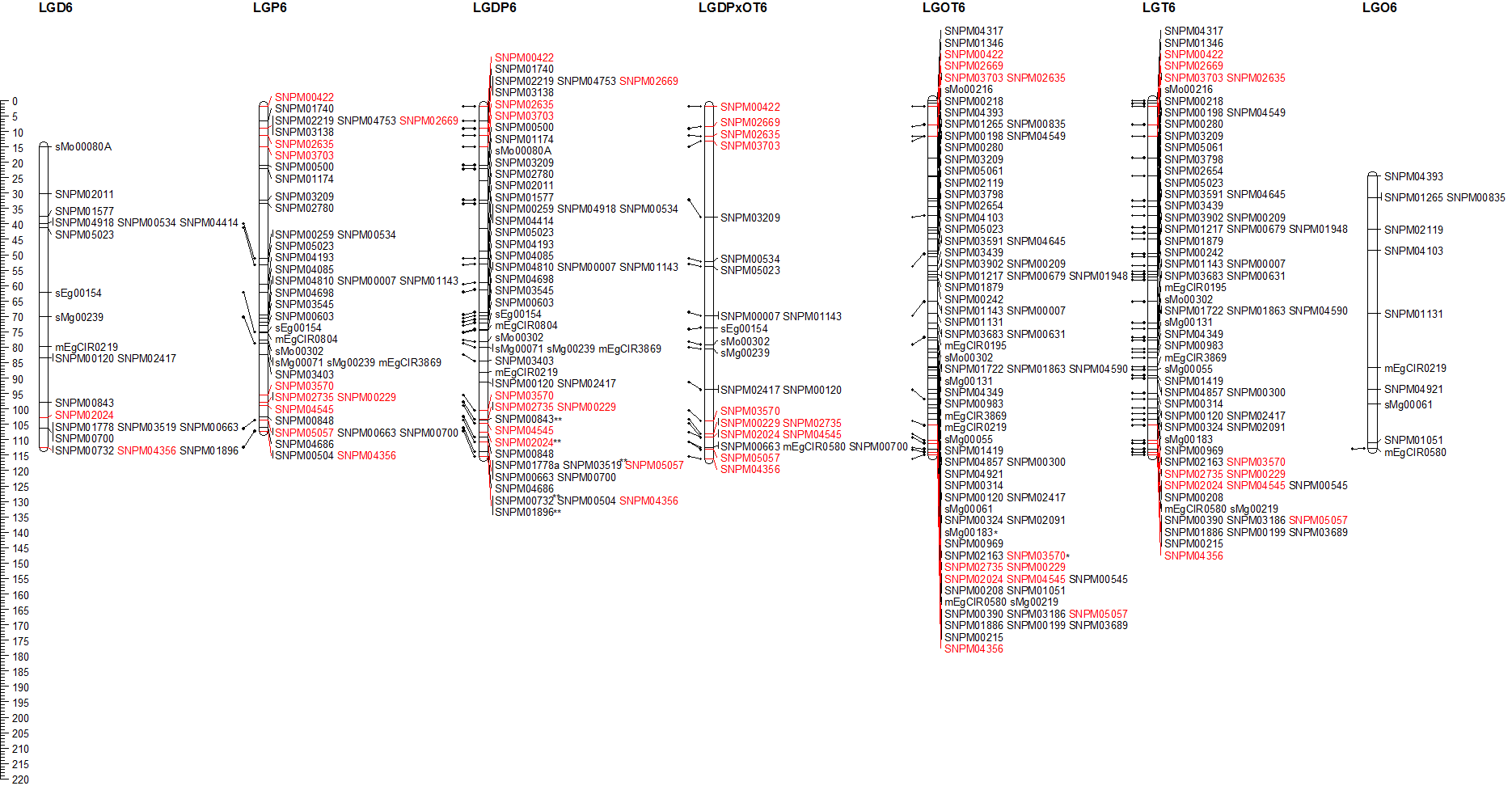

Supplement: Supplementary file 1 — Additional file 1: Alignment of oil palm genetic maps developed for intraspecific ( P2 ) and interspecific ( OxG ) crosses using common markers. The P2 bi-parental integrated (DP), dura parental (D) and pisifera parental (P) maps are shown on the left and OxG bi-parental integrated (OT), E. oleifera parental (O) and tenera parental (T) maps on the right. The integrated map of P2 and OxG is shown at the centre, labeled DPxOT/T. Haldane genetic distance (cM) is indicated by the ruler on the left of the map. Common markers that co-mapped across D, P, DP, DPxOT/T and T are indicated in red. Nomenclature for markers is: SNPM (SNP), mEgCIR (genomic SSR from [8, 23], sEg (E. guineensis EST-SSR), sMg (E. guineensis genomic-SSR), sMo (E. oleifera genomic-SSR), sPSc (SSR developed from E. guineensis scaffold data). Markers showing distorted segregation are marked by *representing significance, viz.,*p < 0.1,**p < 0.05,***p < 0.01,****p < 0.005,*****p < 0.001 and ******p < 0.0005. (ZIP 6 MB) [file 12864_2013_7049_MOESM1_ESM.zip › LG6.tif]

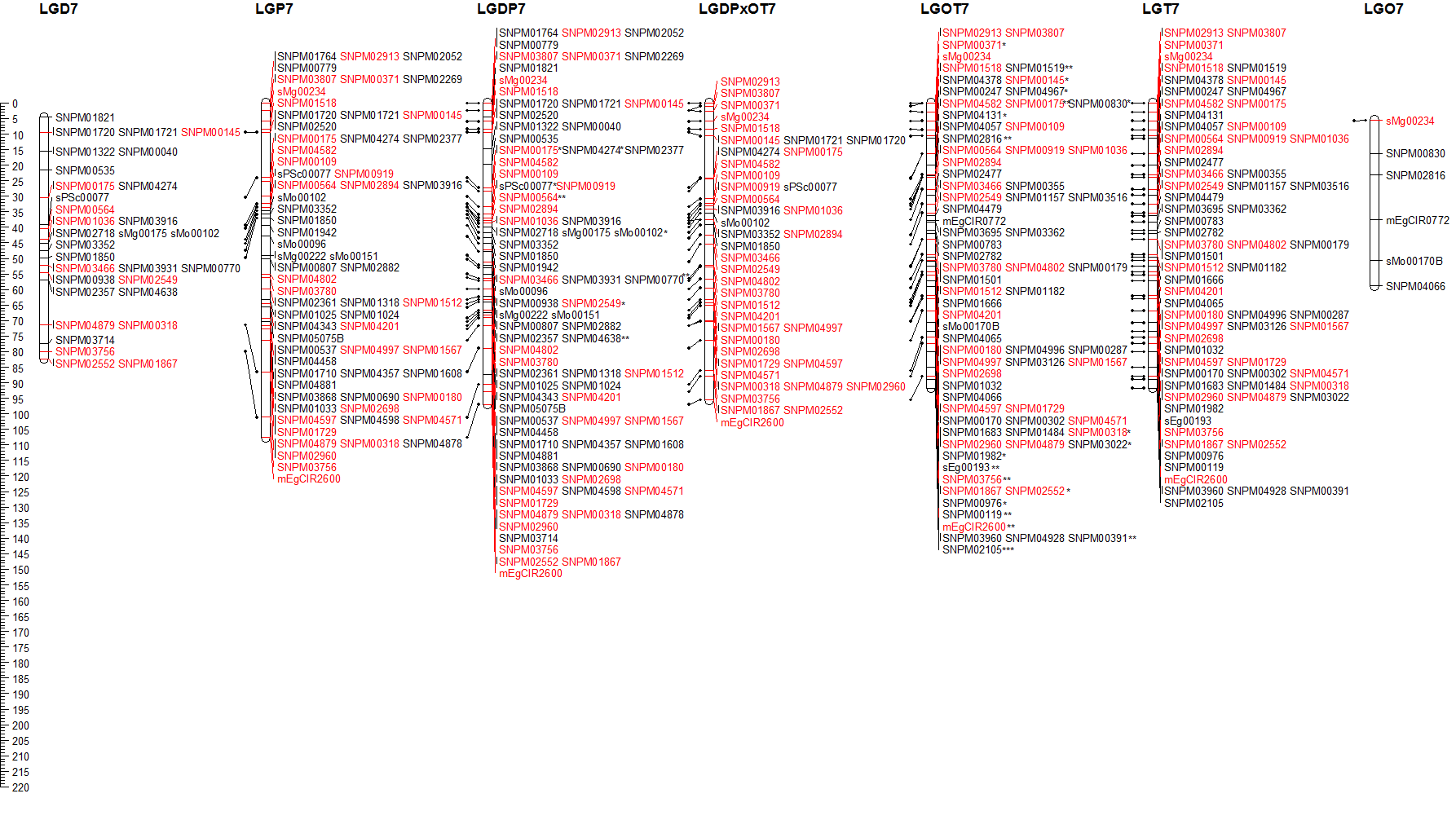

Supplement: Supplementary file 1 — Additional file 1: Alignment of oil palm genetic maps developed for intraspecific ( P2 ) and interspecific ( OxG ) crosses using common markers. The P2 bi-parental integrated (DP), dura parental (D) and pisifera parental (P) maps are shown on the left and OxG bi-parental integrated (OT), E. oleifera parental (O) and tenera parental (T) maps on the right. The integrated map of P2 and OxG is shown at the centre, labeled DPxOT/T. Haldane genetic distance (cM) is indicated by the ruler on the left of the map. Common markers that co-mapped across D, P, DP, DPxOT/T and T are indicated in red. Nomenclature for markers is: SNPM (SNP), mEgCIR (genomic SSR from [8, 23], sEg (E. guineensis EST-SSR), sMg (E. guineensis genomic-SSR), sMo (E. oleifera genomic-SSR), sPSc (SSR developed from E. guineensis scaffold data). Markers showing distorted segregation are marked by *representing significance, viz.,*p < 0.1,**p < 0.05,***p < 0.01,****p < 0.005,*****p < 0.001 and ******p < 0.0005. (ZIP 6 MB) [file 12864_2013_7049_MOESM1_ESM.zip › LG7.tif]

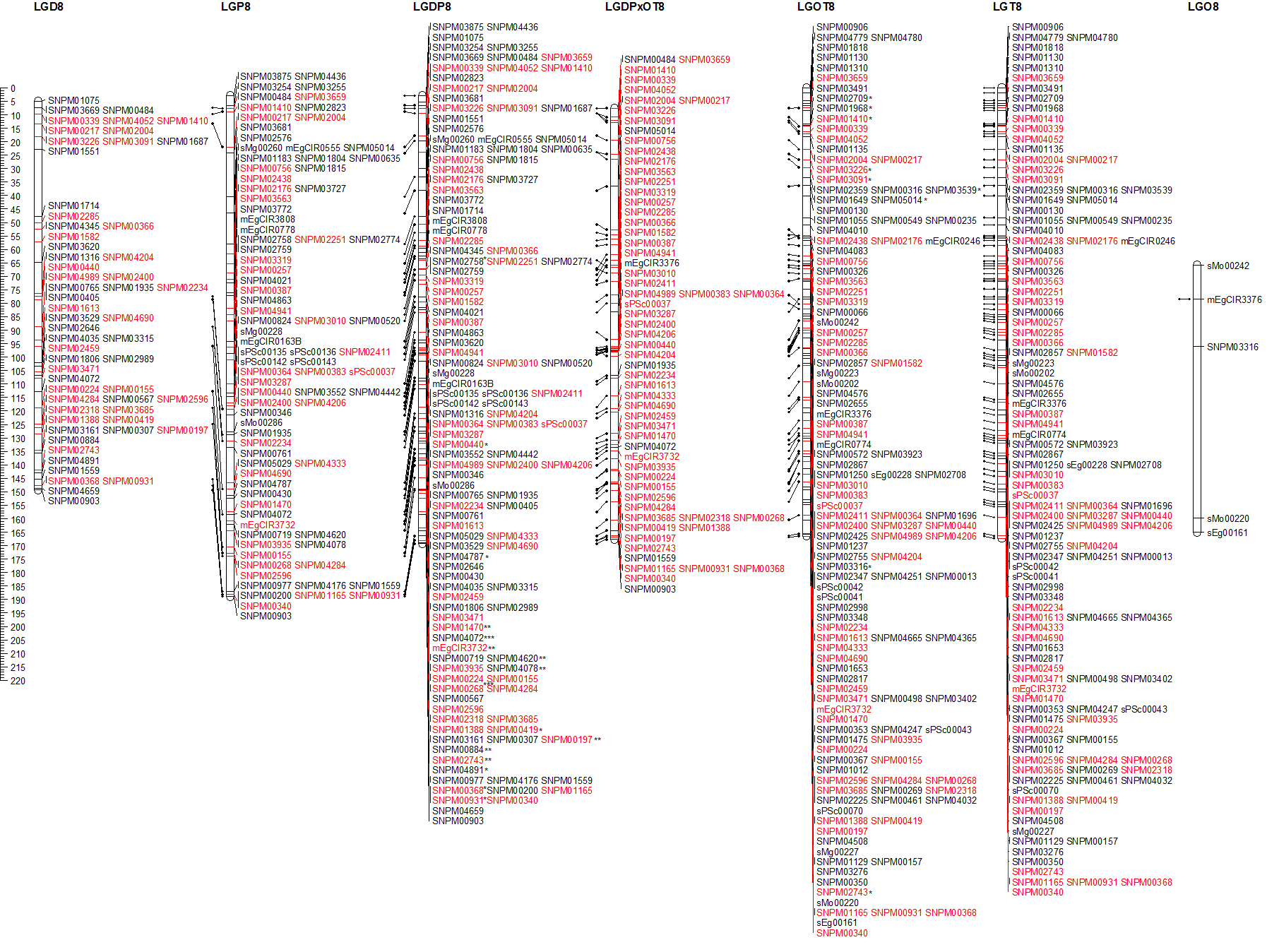

Supplement: Supplementary file 1 — Additional file 1: Alignment of oil palm genetic maps developed for intraspecific ( P2 ) and interspecific ( OxG ) crosses using common markers. The P2 bi-parental integrated (DP), dura parental (D) and pisifera parental (P) maps are shown on the left and OxG bi-parental integrated (OT), E. oleifera parental (O) and tenera parental (T) maps on the right. The integrated map of P2 and OxG is shown at the centre, labeled DPxOT/T. Haldane genetic distance (cM) is indicated by the ruler on the left of the map. Common markers that co-mapped across D, P, DP, DPxOT/T and T are indicated in red. Nomenclature for markers is: SNPM (SNP), mEgCIR (genomic SSR from [8, 23], sEg (E. guineensis EST-SSR), sMg (E. guineensis genomic-SSR), sMo (E. oleifera genomic-SSR), sPSc (SSR developed from E. guineensis scaffold data). Markers showing distorted segregation are marked by *representing significance, viz.,*p < 0.1,**p < 0.05,***p < 0.01,****p < 0.005,*****p < 0.001 and ******p < 0.0005. (ZIP 6 MB) [file 12864_2013_7049_MOESM1_ESM.zip › LG8.tif]

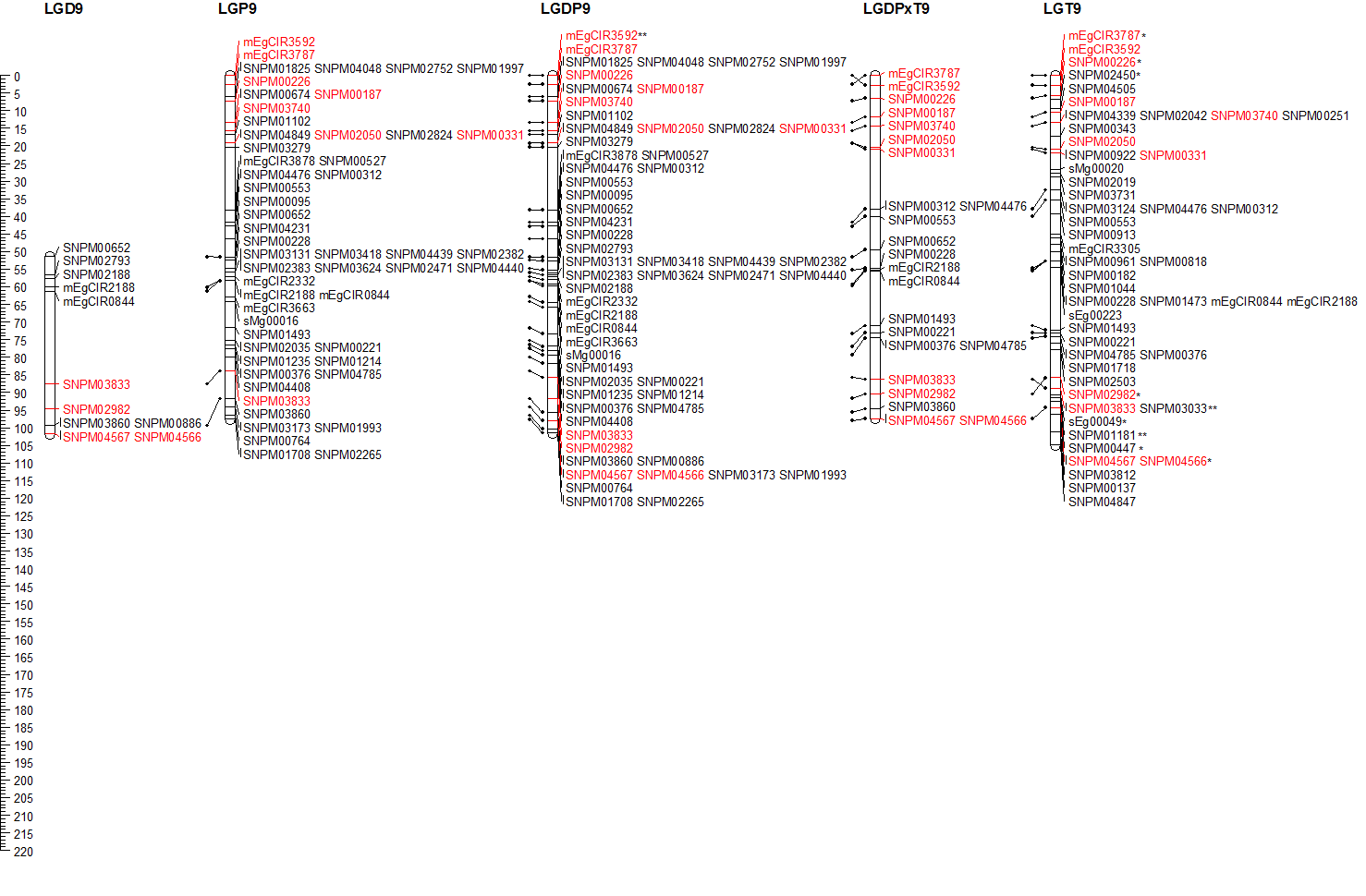

Supplement: Supplementary file 1 — Additional file 1: Alignment of oil palm genetic maps developed for intraspecific ( P2 ) and interspecific ( OxG ) crosses using common markers. The P2 bi-parental integrated (DP), dura parental (D) and pisifera parental (P) maps are shown on the left and OxG bi-parental integrated (OT), E. oleifera parental (O) and tenera parental (T) maps on the right. The integrated map of P2 and OxG is shown at the centre, labeled DPxOT/T. Haldane genetic distance (cM) is indicated by the ruler on the left of the map. Common markers that co-mapped across D, P, DP, DPxOT/T and T are indicated in red. Nomenclature for markers is: SNPM (SNP), mEgCIR (genomic SSR from [8, 23], sEg (E. guineensis EST-SSR), sMg (E. guineensis genomic-SSR), sMo (E. oleifera genomic-SSR), sPSc (SSR developed from E. guineensis scaffold data). Markers showing distorted segregation are marked by *representing significance, viz.,*p < 0.1,**p < 0.05,***p < 0.01,****p < 0.005,*****p < 0.001 and ******p < 0.0005. (ZIP 6 MB) [file 12864_2013_7049_MOESM1_ESM.zip › LG9.tif]

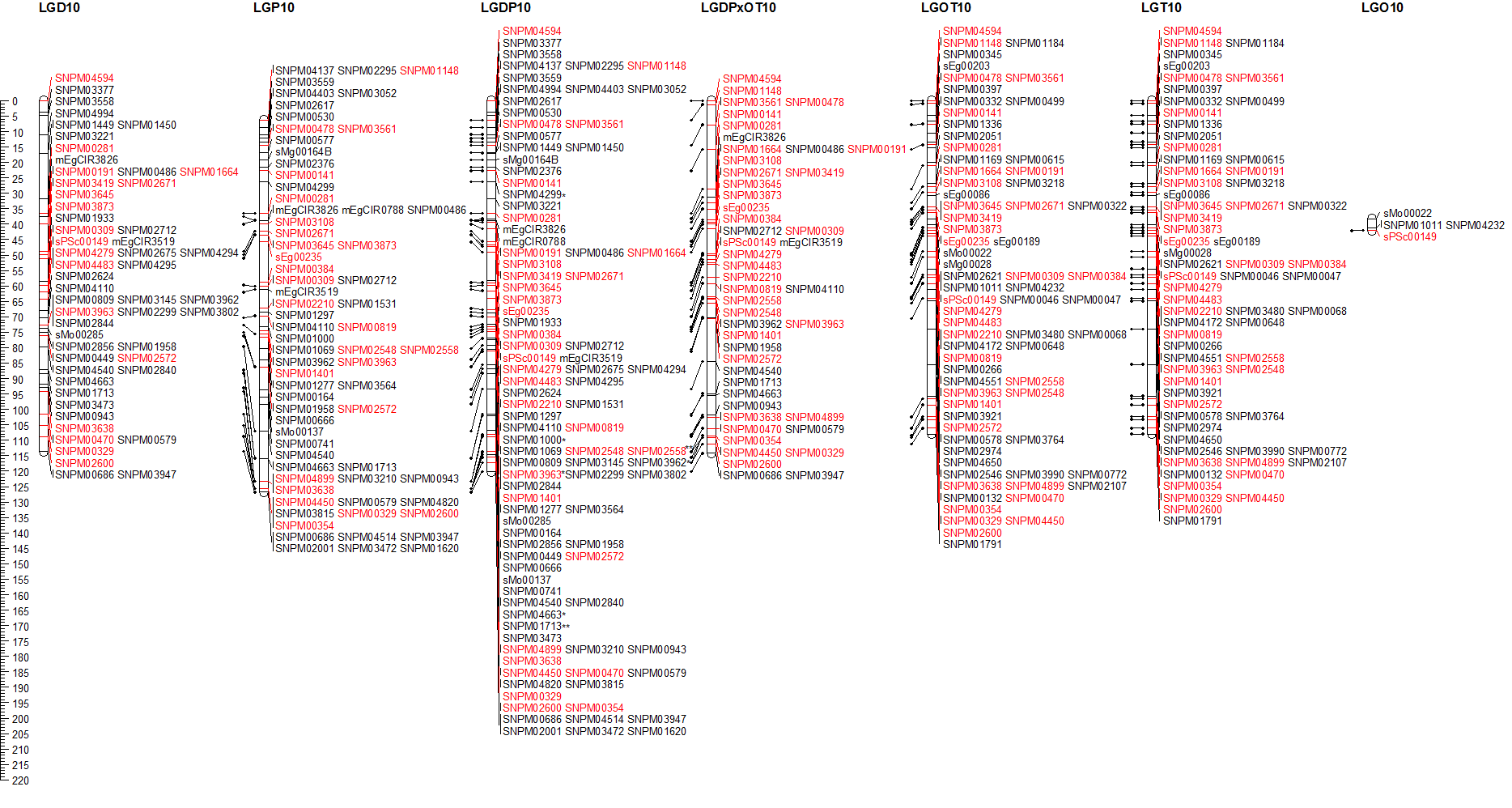

Supplement: Supplementary file 1 — Additional file 1: Alignment of oil palm genetic maps developed for intraspecific ( P2 ) and interspecific ( OxG ) crosses using common markers. The P2 bi-parental integrated (DP), dura parental (D) and pisifera parental (P) maps are shown on the left and OxG bi-parental integrated (OT), E. oleifera parental (O) and tenera parental (T) maps on the right. The integrated map of P2 and OxG is shown at the centre, labeled DPxOT/T. Haldane genetic distance (cM) is indicated by the ruler on the left of the map. Common markers that co-mapped across D, P, DP, DPxOT/T and T are indicated in red. Nomenclature for markers is: SNPM (SNP), mEgCIR (genomic SSR from [8, 23], sEg (E. guineensis EST-SSR), sMg (E. guineensis genomic-SSR), sMo (E. oleifera genomic-SSR), sPSc (SSR developed from E. guineensis scaffold data). Markers showing distorted segregation are marked by *representing significance, viz.,*p < 0.1,**p < 0.05,***p < 0.01,****p < 0.005,*****p < 0.001 and ******p < 0.0005. (ZIP 6 MB) [file 12864_2013_7049_MOESM1_ESM.zip › LG10.tif]

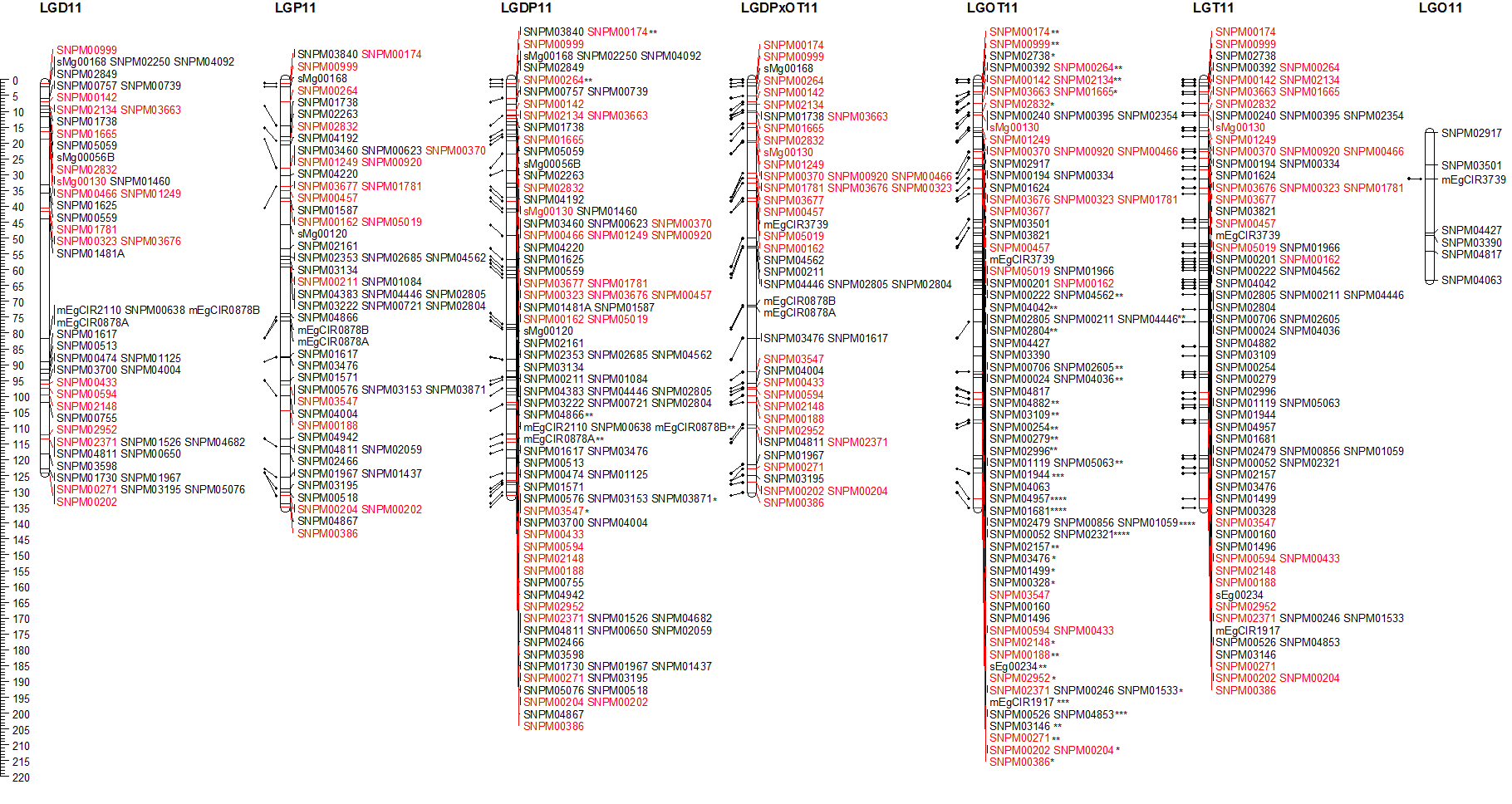

Supplement: Supplementary file 1 — Additional file 1: Alignment of oil palm genetic maps developed for intraspecific ( P2 ) and interspecific ( OxG ) crosses using common markers. The P2 bi-parental integrated (DP), dura parental (D) and pisifera parental (P) maps are shown on the left and OxG bi-parental integrated (OT), E. oleifera parental (O) and tenera parental (T) maps on the right. The integrated map of P2 and OxG is shown at the centre, labeled DPxOT/T. Haldane genetic distance (cM) is indicated by the ruler on the left of the map. Common markers that co-mapped across D, P, DP, DPxOT/T and T are indicated in red. Nomenclature for markers is: SNPM (SNP), mEgCIR (genomic SSR from [8, 23], sEg (E. guineensis EST-SSR), sMg (E. guineensis genomic-SSR), sMo (E. oleifera genomic-SSR), sPSc (SSR developed from E. guineensis scaffold data). Markers showing distorted segregation are marked by *representing significance, viz.,*p < 0.1,**p < 0.05,***p < 0.01,****p < 0.005,*****p < 0.001 and ******p < 0.0005. (ZIP 6 MB) [file 12864_2013_7049_MOESM1_ESM.zip › LG11.tif]

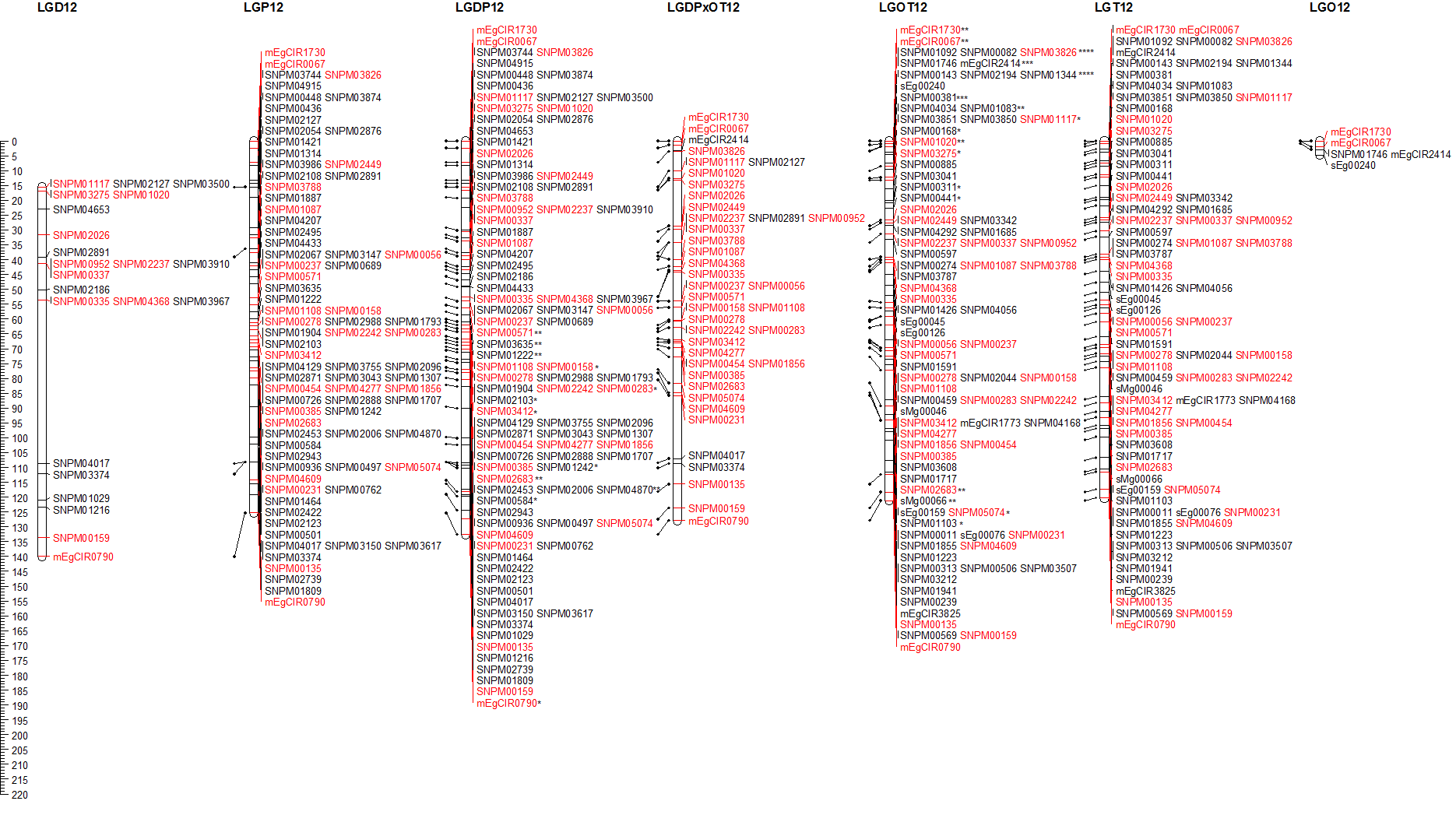

Supplement: Supplementary file 1 — Additional file 1: Alignment of oil palm genetic maps developed for intraspecific ( P2 ) and interspecific ( OxG ) crosses using common markers. The P2 bi-parental integrated (DP), dura parental (D) and pisifera parental (P) maps are shown on the left and OxG bi-parental integrated (OT), E. oleifera parental (O) and tenera parental (T) maps on the right. The integrated map of P2 and OxG is shown at the centre, labeled DPxOT/T. Haldane genetic distance (cM) is indicated by the ruler on the left of the map. Common markers that co-mapped across D, P, DP, DPxOT/T and T are indicated in red. Nomenclature for markers is: SNPM (SNP), mEgCIR (genomic SSR from [8, 23], sEg (E. guineensis EST-SSR), sMg (E. guineensis genomic-SSR), sMo (E. oleifera genomic-SSR), sPSc (SSR developed from E. guineensis scaffold data). Markers showing distorted segregation are marked by *representing significance, viz.,*p < 0.1,**p < 0.05,***p < 0.01,****p < 0.005,*****p < 0.001 and ******p < 0.0005. (ZIP 6 MB) [file 12864_2013_7049_MOESM1_ESM.zip › LG12.tif]

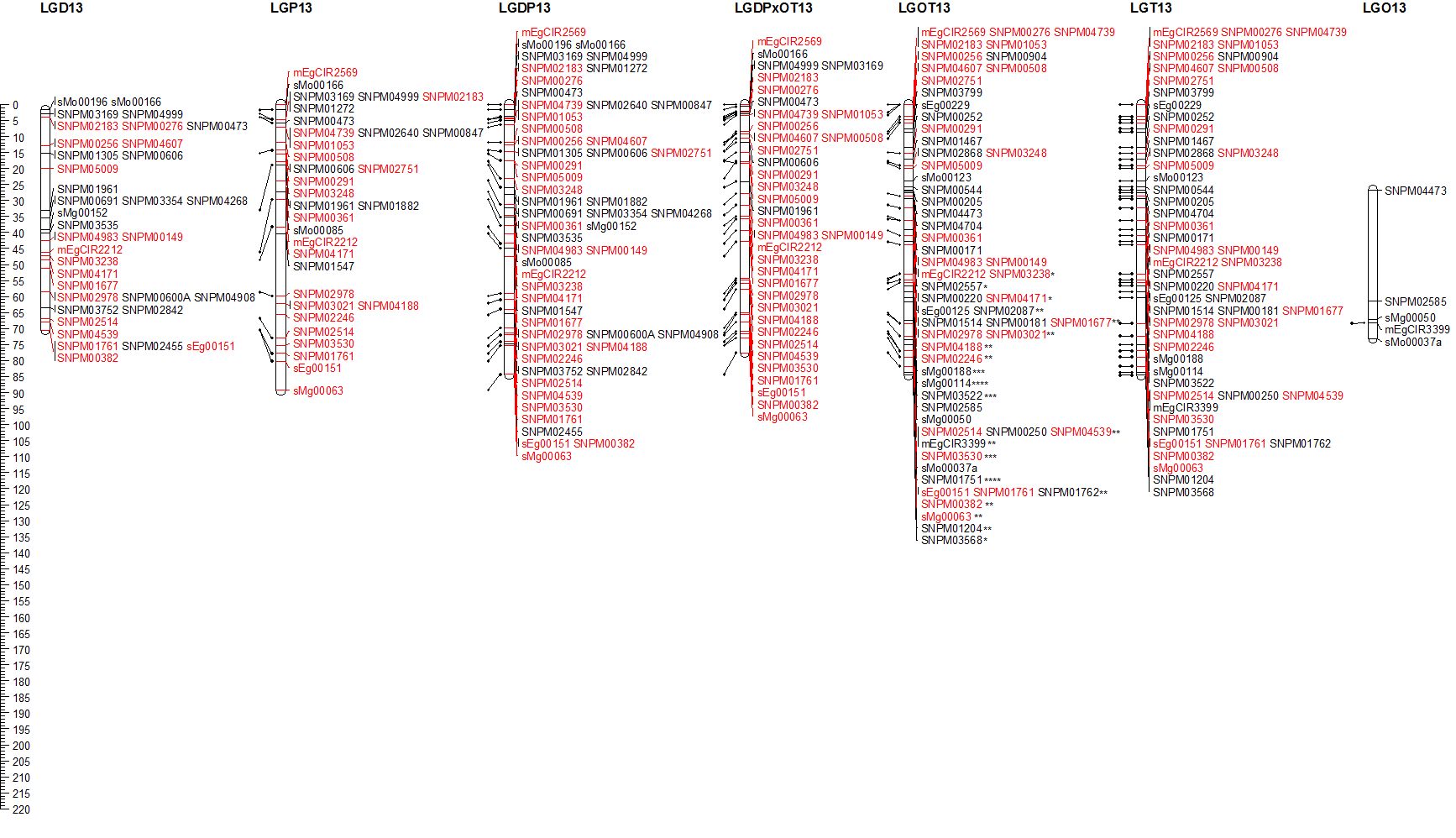

Supplement: Supplementary file 1 — Additional file 1: Alignment of oil palm genetic maps developed for intraspecific ( P2 ) and interspecific ( OxG ) crosses using common markers. The P2 bi-parental integrated (DP), dura parental (D) and pisifera parental (P) maps are shown on the left and OxG bi-parental integrated (OT), E. oleifera parental (O) and tenera parental (T) maps on the right. The integrated map of P2 and OxG is shown at the centre, labeled DPxOT/T. Haldane genetic distance (cM) is indicated by the ruler on the left of the map. Common markers that co-mapped across D, P, DP, DPxOT/T and T are indicated in red. Nomenclature for markers is: SNPM (SNP), mEgCIR (genomic SSR from [8, 23], sEg (E. guineensis EST-SSR), sMg (E. guineensis genomic-SSR), sMo (E. oleifera genomic-SSR), sPSc (SSR developed from E. guineensis scaffold data). Markers showing distorted segregation are marked by *representing significance, viz.,*p < 0.1,**p < 0.05,***p < 0.01,****p < 0.005,*****p < 0.001 and ******p < 0.0005. (ZIP 6 MB) [file 12864_2013_7049_MOESM1_ESM.zip › LG13.tif]

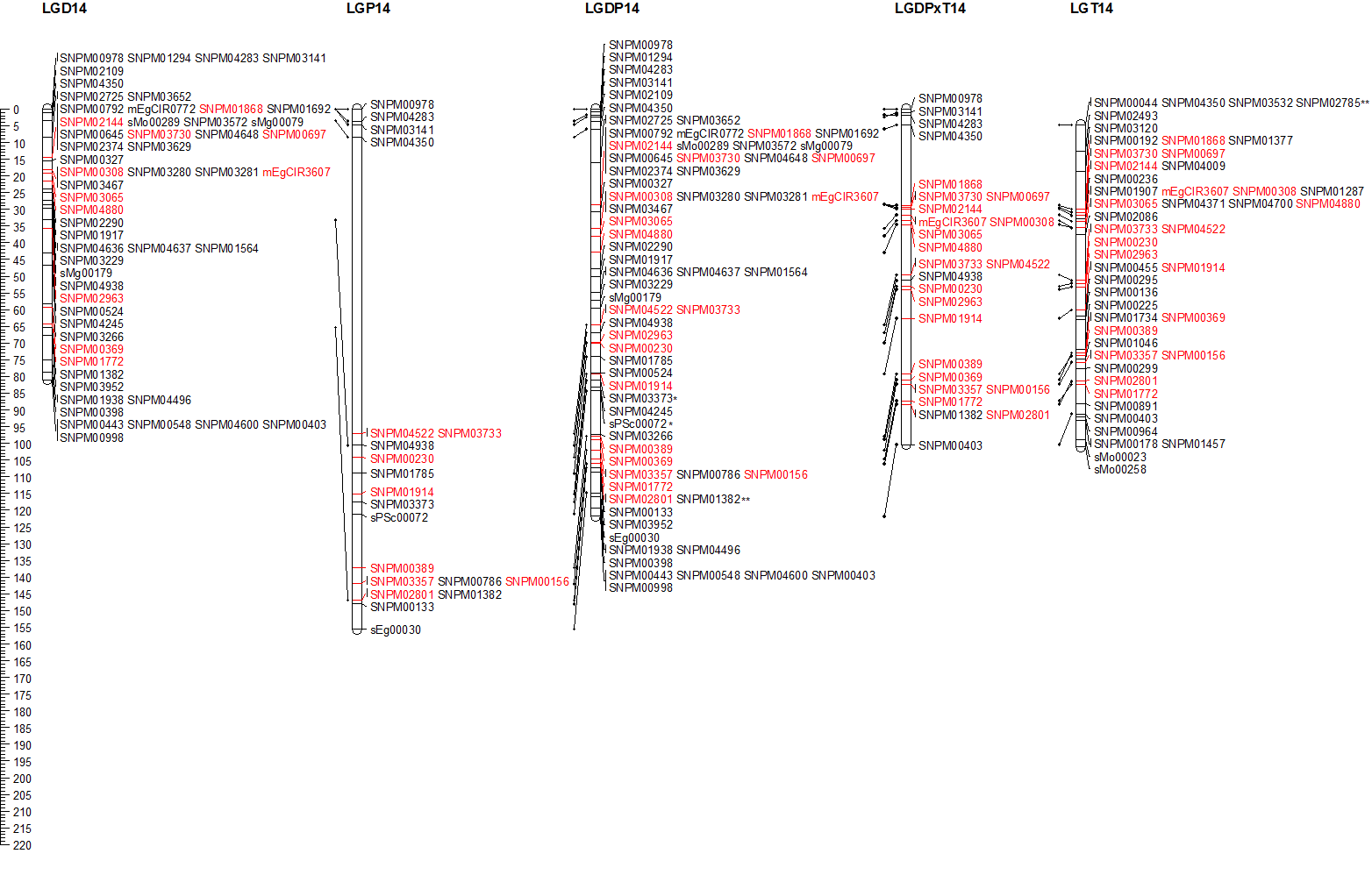

Supplement: Supplementary file 1 — Additional file 1: Alignment of oil palm genetic maps developed for intraspecific ( P2 ) and interspecific ( OxG ) crosses using common markers. The P2 bi-parental integrated (DP), dura parental (D) and pisifera parental (P) maps are shown on the left and OxG bi-parental integrated (OT), E. oleifera parental (O) and tenera parental (T) maps on the right. The integrated map of P2 and OxG is shown at the centre, labeled DPxOT/T. Haldane genetic distance (cM) is indicated by the ruler on the left of the map. Common markers that co-mapped across D, P, DP, DPxOT/T and T are indicated in red. Nomenclature for markers is: SNPM (SNP), mEgCIR (genomic SSR from [8, 23], sEg (E. guineensis EST-SSR), sMg (E. guineensis genomic-SSR), sMo (E. oleifera genomic-SSR), sPSc (SSR developed from E. guineensis scaffold data). Markers showing distorted segregation are marked by *representing significance, viz.,*p < 0.1,**p < 0.05,***p < 0.01,****p < 0.005,*****p < 0.001 and ******p < 0.0005. (ZIP 6 MB) [file 12864_2013_7049_MOESM1_ESM.zip › LG14.tif]

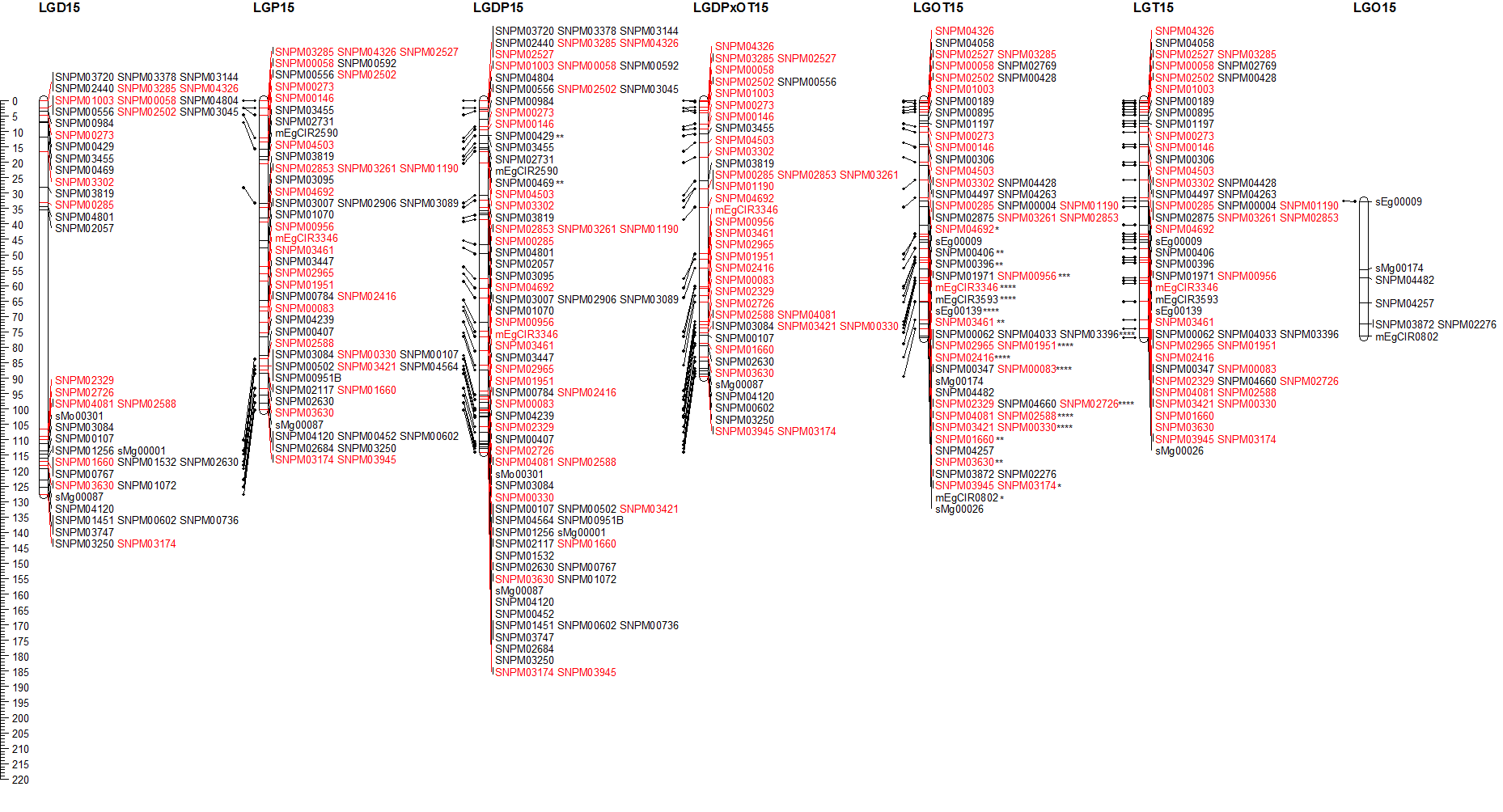

Supplement: Supplementary file 1 — Additional file 1: Alignment of oil palm genetic maps developed for intraspecific ( P2 ) and interspecific ( OxG ) crosses using common markers. The P2 bi-parental integrated (DP), dura parental (D) and pisifera parental (P) maps are shown on the left and OxG bi-parental integrated (OT), E. oleifera parental (O) and tenera parental (T) maps on the right. The integrated map of P2 and OxG is shown at the centre, labeled DPxOT/T. Haldane genetic distance (cM) is indicated by the ruler on the left of the map. Common markers that co-mapped across D, P, DP, DPxOT/T and T are indicated in red. Nomenclature for markers is: SNPM (SNP), mEgCIR (genomic SSR from [8, 23], sEg (E. guineensis EST-SSR), sMg (E. guineensis genomic-SSR), sMo (E. oleifera genomic-SSR), sPSc (SSR developed from E. guineensis scaffold data). Markers showing distorted segregation are marked by *representing significance, viz.,*p < 0.1,**p < 0.05,***p < 0.01,****p < 0.005,*****p < 0.001 and ******p < 0.0005. (ZIP 6 MB) [file 12864_2013_7049_MOESM1_ESM.zip › LG15.tif]

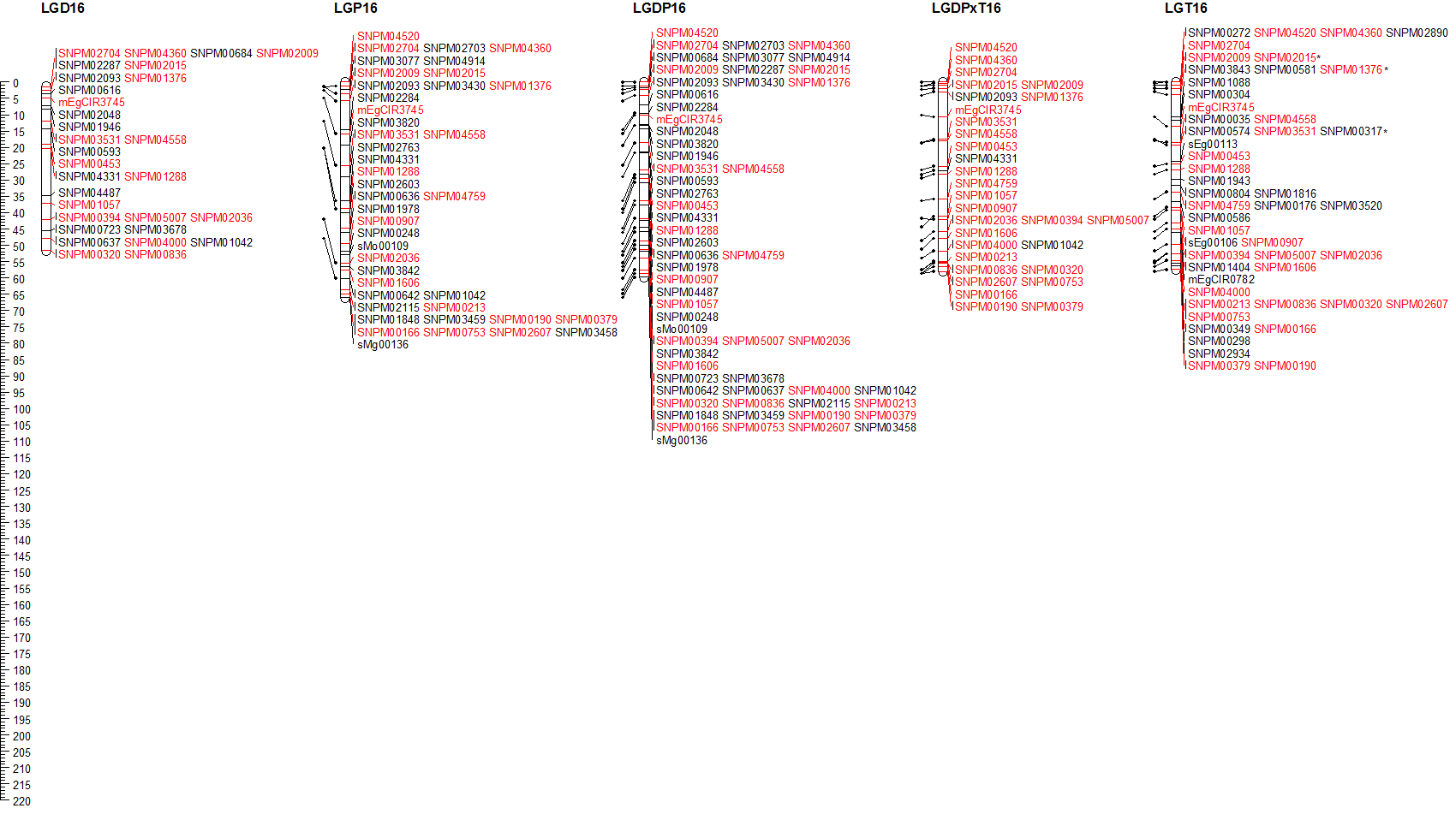

Supplement: Supplementary file 1 — Additional file 1: Alignment of oil palm genetic maps developed for intraspecific ( P2 ) and interspecific ( OxG ) crosses using common markers. The P2 bi-parental integrated (DP), dura parental (D) and pisifera parental (P) maps are shown on the left and OxG bi-parental integrated (OT), E. oleifera parental (O) and tenera parental (T) maps on the right. The integrated map of P2 and OxG is shown at the centre, labeled DPxOT/T. Haldane genetic distance (cM) is indicated by the ruler on the left of the map. Common markers that co-mapped across D, P, DP, DPxOT/T and T are indicated in red. Nomenclature for markers is: SNPM (SNP), mEgCIR (genomic SSR from [8, 23], sEg (E. guineensis EST-SSR), sMg (E. guineensis genomic-SSR), sMo (E. oleifera genomic-SSR), sPSc (SSR developed from E. guineensis scaffold data). Markers showing distorted segregation are marked by *representing significance, viz.,*p < 0.1,**p < 0.05,***p < 0.01,****p < 0.005,*****p < 0.001 and ******p < 0.0005. (ZIP 6 MB) [file 12864_2013_7049_MOESM1_ESM.zip › LG16.tif]
