# Supplementary material for: High density SNP and SSR-based genetic maps of two independent oil palm hybrids
Source: BMC Genomics. 2014 Apr 27;15(1):309. doi: 10.1186/1471-2164-15-309 (PMC4234488; doi:10.1186/1471-2164-15-309)
Supplement: Supplementary file 2 — Additional file 2: Information on P2 (D, P and DP) and OxG (O, T and OT) genetic maps as well as the cross-population integrated map (DPxOT/T). (DOCX 62 KB) [file 12864_2013_7049_MOESM2_ESM.docx]

**Additional file 1: Information on P2 (D, P and DP) and OxG (O, T and OT) genetic maps as well as the cross-population integrated map (DPxOT/T).**

| LG | Map information | P2 population | | | OxG population | | | Cross-population Integrated map |
| --- | --- | --- | --- | --- | --- | --- | --- | --- |
|  |  | D | P | DP | O | T | OT | DPxOT/T |
| 1 | Number of markers | 62 | 83 | 115 | 4 | 94 | 97 | 60 |
|  | Map length (cM) | 91.7 | 158.9 | 151.1 | 13.1 | 133.3 | 133.3 | 131.4 |
|  | Average distance (cM) | 1.5 | 1.9 | 1.3 | 3.3 | 1.4 | 1.4 | 2.2 |
|  | Maximum gap size (cM) | 8.8 | 16.1 | 16.1 | 10.2 | 6.9 | 6.9 | 19.1 |
|  |  |  |  |  |  |  |  |  |
| 2 | Number of markers | 70 | 59 | 108 | - | 62 | - | 44 |
|  | Map length (cM) | 91 | 114.6 | 104.3 | - | 110.9 | - | 113.7 |
|  | Average distance (cM) | 1.3 | 1.9 | 1.0 | - | 1.8 | - | 2.6 |
|  | Maximum gap size (cM) | 11.6 | 14.6 | 8.7 | - | 10.3 | - | 24.2 |
|  |  |  |  |  |  |  |  |  |
| 3 | Number of markers | 32 | 41 | 61 | - | 42 | - | 28 |
|  | Map length (cM) | 67.4 | 79.5 | 80.5 | - | 80 | - | 73.4 |
|  | Average distance (cM) | 2.1 | 1.9 | 1.3 | - | 1.9 | - | 2.6 |
|  | Maximum gap size (cM) | 11.0 | 10.9 | 9.6 | - | 12.6 | - | 8.0 |
|  |  |  |  |  |  |  |  |  |
| 4 | Number of markers | 32 | 125 | 146 | 11 | 140 | 150 | 58 |
|  | Map length (cM) | 103.9 | 214.6 | 220.2 | 71.5 | 216.8 | 216.8 | 202.1 |
|  | Average distance (cM) | 3.2 | 1.7 | 1.5 | 6.5 | 1.5 | 1.4 | 3.5 |
|  | Maximum gap size (cM) | 48.5 | 14.6 | 14.6 | 18.9 | 10.2 | 10.2 | 19.6 |
|  |  |  |  |  |  |  |  |  |
| 5 | Number of markers | 14 | 15 | 27 | - | 24 | - | 10 |
|  | Map length (cM) | 56.4 | 60.9 | 68.4 | - | 60.6 | - | 64.2 |
|  | Average distance (cM) | 4.0 | 4.0 | 2.5 | - | 2.5 | - | 6.4 |
|  | Maximum gap size (cM) | 14.6 | 21.1 | 14.6 | - | 12.6 | - | 13.5 |
|  |  |  |  |  |  |  |  |  |
| 6 | Number of markers | 21 | 41 | 55 | 11 | 68 | 78 | 24 |
|  | Map length (cM) | 97.6 | 105.3 | 113.6 | 88.3 | 114.9 | 114.9 | 114.4 |
|  | Average distance (cM) | 4.6 | 2.6 | 2.1 | 8.0 | 1.7 | 1.5 | 4.8 |
|  | Maximum gap size (cM) | 20.9 | 17.7 | 9.2 | 20.3 | 8.0 | 7.0 | 24.7 |
|  |  |  |  |  |  |  |  |  |
| 7 | Number of markers | 31 | 65 | 82 | 6 | 70 | 75 | 41 |
|  | Map length (cM) | 77.7 | 107.5 | 97 | 53.2 | 91.7 | 91.7 | 95.5 |
|  | Average distance (cM) | 2.5 | 1.6 | 1.2 | 8.9 | 1.3 | 1.2 | 2.3 |
|  | Maximum gap size (cM) | 14.6 | 14.6 | 8.4 | 14.3 | 4.9 | 4.9 | 13.5 |
|  |  |  |  |  |  |  |  |  |
| 8 | Number of markers | 58 | 85 | 127 | 5 | 127 | 131 | 62 |
|  | Map length (cM) | 144.3 | 185.9 | 166.3 | 99.2 | 167.3 | 166.5 | 160.1 |
|  | Average distance (cM) | 2.5 | 2.2 | 1.3 | 19.8 | 1.3 | 1.3 | 2.6 |
|  | Maximum gap size (cM) | 24.8 | 16.2 | 9.6 | 63.6 | 8.1 | 8.1 | 16.1 |
|  |  |  |  |  |  |  |  |  |
| 9 | Number of markers | 11 | 53 | 59 | - | 49 | - | 23 |
|  | Map length (cM) | 50.3 | 97.6 | 101.4 | - | 104.9 | - | 97.4 |
|  | Average distance (cM) | 4.6 | 1.8 | 1.7 | - | 2.1 | - | 4.2 |
|  | Maximum gap size (cM) | 26.3 | 17.8 | 17.8 | - | 17.6 | - | 17.0 |
|  |  |  |  |  |  |  |  |  |
| 10 | Number of markers | 53 | 68 | 97 | 4 | 69 | 72 | 47 |
|  | Map length (cM) | 113.6 | 120.5 | 120.2 | 3.8 | 108 | 108 | 114.2 |
|  | Average distance (cM) | 2.1 | 1.8 | 1.2 | 1.0 | 1.6 | 1.5 | 2.4 |
|  | Maximum gap size (cM) | 14.6 | 13.1 | 9.1 | 2.9 | 11.4 | 11.4 | 13.9 |
|  |  |  |  |  |  |  |  |  |
| 11 | Number of markers | 52 | 59 | 97 | 7 | 82 | 88 | 47 |
|  | Map length (cM) | 123 | 135 | 131.3 | 46.5 | 135.2 | 135.2 | 130.4 |
|  | Average distance (cM) | 2.4 | 2.3 | 1.4 | 6.6 | 1.6 | 1.5 | 2.8 |
|  | Maximum gap size (cM) | 37.6 | 14.6 | 14.6 | 17.0 | 10.3 | 10.3 | 18.0 |
|  |  |  |  |  |  |  |  |  |
| 12 | Number of markers | 22 | 79 | 96 | 5 | 84 | 86 | 40 |
|  | Map length (cM) | 124.4 | 125.3 | 132.6 | 4.8 | 120.2 | 121.2 | 127.9 |
|  | Average distance (cM) | 5.7 | 1.6 | 1.4 | 1.0 | 1.4 | 1.4 | 3.2 |
|  | Maximum gap size (cM) | 55.0 | 10.2 | 10.0 | 2.0 | 9.8 | 9.9 | 21.2 |
|  |  |  |  |  |  |  |  |  |
| 13 | Number of markers | 35 | 32 | 54 | 5 | 56 | 60 | 36 |
|  | Map length (cM) | 68.8 | 89.2 | 84.3 | 46.4 | 84.6 | 84.6 | 77.6 |
|  | Average distance (cM) | 2.0 | 2.8 | 1.6 | 9.3 | 1.5 | 1.4 | 2.2 |
|  | Maximum gap size (cM) | 13.0 | 19.5 | 11.7 | 34.7 | 9.1 | 9.1 | 11.5 |
|  |  |  |  |  |  |  |  |  |
| 14 | Number of markers | 54 | 20 | 68 | - | 49 | - | 26 |
|  | Map length (cM) | 81 | 155.6 | 121.8 | - | 96.2 | - | 100.4 |
|  | Average distance (cM) | 1.5 | 7.8 | 1.8 | - | 2.0 | - | 3.9 |
|  | Maximum gap size (cM) | 11.6 | 88.7 | 13.1 | - | 13.8 | - | 24.0 |
|  |  |  |  |  |  |  |  |  |
| 15 | Number of markers | 45 | 54 | 80 | 7 | 56 | 62 | 42 |
|  | Map length (cM) | 127.7 | 100.3 | 114 | 43.7 | 76.9 | 76.9 | 89.5 |
|  | Average distance (cM) | 2.8 | 1.9 | 1.4 | 6.2 | 1.4 | 1.2 | 2.1 |
|  | Maximum gap size (cM) | 71.1 | 12.9 | 10.6 | 22.0 | 5.9 | 5.9 | 15.1 |
|  |  |  |  |  |  |  |  |  |
| 16 | Number of markers | 30 | 42 | 59 | - | 49 | - | 30 |
|  | Map length (cM) | 50.2 | 66 | 59.8 | - | 57.4 | - | 58 |
|  | Average distance (cM) | 1.7 | 1.6 | 1.0 | - | 1.2 | - | 1.9 |
|  | Maximum gap size (cM) | 14.6 | 8.8 | 5.6 | - | 6.9 | - | 7.7 |
