# Supplementary material for: High density SNP and SSR-based genetic maps of two independent oil palm hybrids
Source: BMC Genomics. 2014 Apr 27;15(1):309. doi: 10.1186/1471-2164-15-309 (PMC4234488; doi:10.1186/1471-2164-15-309)
Supplement: Supplementary file 3 — Additional file 3: A closer view of comparative linkage groups (LGs) of dura (D), pisifera (P), E. oleifera (O) and tenera (T) plotted with Circos [[60]]. LGs 1 – 16 are scaled in Haldane genetic distance (cM) and represented by different color coded lines. (DOCX 827 KB) [file 12864_2013_7049_MOESM3_ESM.docx]

**Additional file 2. A closer view of comparative linkage groups (LGs) of *dura* (D), *pisifera* (P), *E. oleifera* (O), *tenera* (T) and the integrated map (DPxOT/T) plotted with Circos [60].** LGs 1 – 16 are scaled in Haldane genetic distance (cM) and represented by different color coded lines.

**
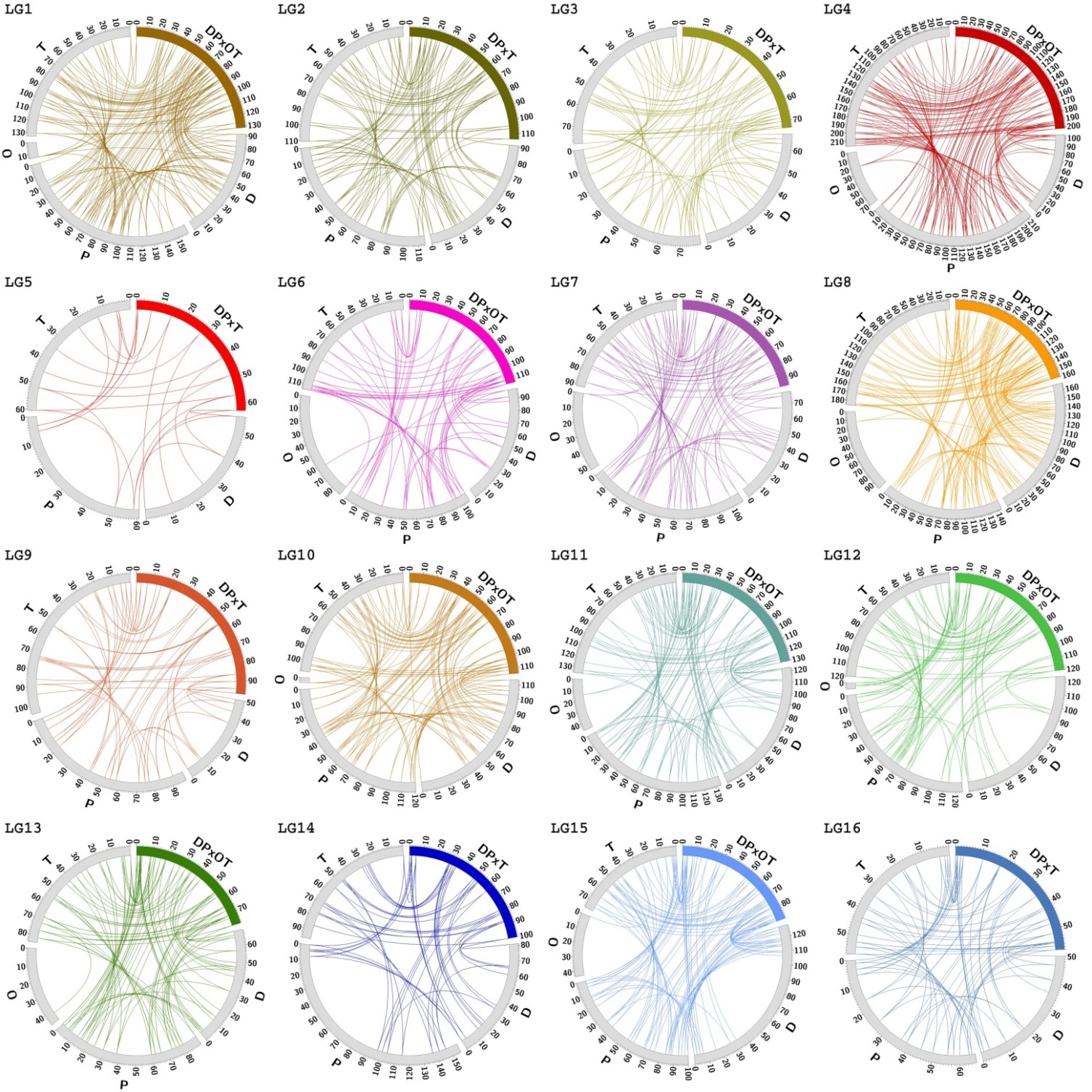
**
