# Supplementary material for: High density SNP and SSR-based genetic maps of two independent oil palm hybrids
Source: BMC Genomics. 2014 Apr 27;15(1):309. doi: 10.1186/1471-2164-15-309 (PMC4234488; doi:10.1186/1471-2164-15-309)
Supplement: Supplementary file 4 — Additional file 4: Comparative linkage groups (LGs) between the current maps and previously published oil palm maps. The P2 integrated map (DP) and OxG integrated map (OT/T) are aligned with maps developed by Billotte et al. [8], Singh et al. [9], Ting et al. [17] and Seng et al. [24] based on common RFLP and SSR markers. (DOCX 664 KB) [file 12864_2013_7049_MOESM4_ESM.docx]

**Additional file 3. Comparative linkage groups (LGs) between the current maps and previously published oil palm maps.** The P2 integrated map (DP) and OxG integrated map (OT/T) are aligned with maps developed by Billotte *et al.* [8], Singh *et al*. [9], Ting *et al.* [17] and Seng *et al.* [24] based on common RFLP and SSR markers.
